# Integrated Bioinformatics and Multi-Omics Analysis of ZBTB40 Expression, Prognostic Relevance, and Regulatory Networks in Hepatocellular Carcinoma

**DOI:** 10.3390/medicina62071244

**Published:** 2026-06-27

**Authors:** Tae-Young Kim, Jae-Hee Park, Yong Wook Jung, Jae-Ho Lee, Jongwan Kim

**Affiliations:** 1 Department of Anatomy, School of Medicine, Keimyung University, Daegu 42601, Republic of Korea; kimtae02@dsmc.or.kr; 2 Department of Paramedicine, Keimyung College University, Daegu 42601, Republic of Korea; cpr8282@kmcu.ac.kr; 3 Department of Anatomy, College of Medicine, Dongguk University, Gyeongju 38066, Republic of Korea; jungyw@dongguk.ac.kr

**Keywords:** HCC, ZBTB40, prognostic biomarker, ceRNA, DNA methylation

## Abstract

*Background and Objectives:* Identifying regulatory genes that integrate epigenetic, transcriptional, immune, and non-coding RNA networks may improve prognostic stratification in hepatocellular carcinoma (HCC). ZBTB40 is a poorly characterized transcription factor whose clinical relevance and multi-layered regulatory role in HCC remain unclear. This study systematically investigated the prognostic significance, molecular regulatory networks, and toxicogenomic interactions of ZBTB40 in HCC. *Materials and Methods:* Comprehensive multi-omics analyses were conducted utilizing TCGA-HCC datasets and various public bioinformatics platforms. We systematically evaluated ZBTB40 expression patterns, survival outcomes, clinicopathological associations, DNA methylation status, immune cell infiltration, and competing endogenous RNA (ceRNA) networks. Additionally, chemical–gene interactions were analyzed using the Comparative Toxicogenomics Database (CTD). *Results:* ZBTB40 was significantly overexpressed in HCC, closely correlating with advanced clinicopathological features and poor survival outcomes. This upregulation was significantly associated with promoter hypomethylation. Furthermore, ZBTB40 expression was associated with specific immune infiltration patterns. A ZBTB40-centered ceRNA network identified key regulatory miRNAs, including miR-24-3p, miR-34a-5p, miR-132-3p, and miR-222-3p, along with prognostically relevant lncRNAs and circRNAs. CTD analysis identified 39 key chemical modulators of ZBTB40 (e.g., sorafenib, aflatoxin B1) and revealed RNF13 and CHD3 as functionally related genes sharing substantial chemical interaction profiles. Functional analyses suggested ZBTB40’s involvement in chromatin remodeling, the cell cycle, and immune-related pathways. *Conclusions:* ZBTB40 expression is associated with multi-layered molecular features involving epigenetic, post-transcriptional, immune-related, and toxicogenomic signatures in HCC.

## 1. Introduction

Hepatocellular carcinoma (HCC) is the most common malignancy of the liver and remains one of the leading causes of cancer-related mortality worldwide [1,2,3]. Despite significant advances in surgical resection, locoregional therapy, and systemic treatments, including molecular targeted therapy and immune checkpoint inhibitors, patients with advanced HCC have a poor prognosis [4,5,6]. High rates of recurrence, therapeutic resistance, and marked molecular heterogeneity contribute to the limited long-term survival of patients with HCC [7,8]. Therefore, the identification of reliable molecular biomarkers that reflect tumor biology, predict prognosis, and guide therapeutic strategies is urgently required [9,10,11]. In particular, a deeper understanding of the intricate interplay between diverse etiological factors and the underlying molecular landscapes is essential to overcome the current clinical challenges [12]. Furthermore, clarifying how these multifaceted drivers dictate tumor progression may provide a robust framework for developing more precise diagnostic and therapeutic interventions [12,13].

HCC is characterized by extensive molecular heterogeneity arising from diverse etiologies, including viral infection, metabolic dysfunction, and chronic inflammation [1,14]. Together, these factors drive complex alterations in genetic, epigenetic, and transcriptional programs that determine tumor aggressiveness, immune evasion, and therapeutic resistance [15,16]. Large-scale integrative studies have demonstrated that dysregulation of transcription factors and chromatin-associated proteins plays a central role in controlling oncogenic signaling networks, DNA damage responses, and cellular plasticity in HCC [17,18]. Moreover, epigenetic modifications, such as DNA methylation and histone remodeling, dynamically regulate gene expression [19,20] and shape the immune microenvironment, influencing immune surveillance and immunotherapy response [21,22]. Thus, the intrinsic biological complexity of HCC highlights the importance of advancing integrative strategies for risk stratification and prognostic modeling [23,24]. Current clinical biomarkers, including serum AFP and radiologic criteria, provide valuable clinical information; however, their performance may be limited in certain contexts, particularly in early-stage detection and recurrence prediction after curative treatment [25,26]. Furthermore, intertumoral and intratumoral heterogeneity contributes to variability in therapeutic responses, underscoring the need for more integrative approaches to individualized prognostic assessment [27].

Building on these advances, the systematic investigation of large-scale transcriptomic and clinicogenomic datasets has enabled the identification of regulatory genes with potential relevance to tumor biology and patient outcomes in HCC [18,28]. In our previous study, we used integrative bioinformatics analyses incorporating gene expression profiles and survival data and prioritized candidate upstream regulators that may contribute to prognostic heterogeneity [29]. Among these candidates, the Zinc finger and BTB domain-containing protein 40 (ZBTB40), a member of the ZBTB transcription factor family, emerged as a gene of interest. Although the biological functions of ZBTB40 remain incompletely characterized, members of the ZBTB family have been widely implicated in transcriptional regulation, chromatin remodeling, cellular differentiation, and tumor progression. Emerging evidence suggests that dysregulation of ZBTB family proteins may influence cell proliferation, apoptosis, epithelial–mesenchymal transition, and immune-related pathways in multiple malignancies. Given these observations, ZBTB40 may represent a previously underexplored regulatory factor with potential relevance to tumor behavior and patient outcomes in HCC [30,31].

The zinc finger and BTB/POZ (ZBTB) family comprises a large group of transcriptional regulators characterized by an N-terminal BTB/POZ domain and C-terminal zinc finger motifs that enable protein–protein interactions (PPIs) and sequence-specific DNA binding, thereby modulating gene expression through recruitment of chromatin-modifying co-regulator complexes [32,33]. Functionally, ZBTB proteins participate in cellular development and immune regulation and have been implicated in cancer biology in a context-dependent manner. Through transcriptional and chromatin-associated regulatory mechanisms, ZBTB family proteins have been linked to pathways governing cell cycle progression, apoptosis, cellular plasticity, and tumor–immune interactions, suggesting that dysregulation of these regulators may contribute to oncogenic phenotypes [34,35]. In addition to protein-coding genes, non-coding RNAs (ncRNAs) constitute a major regulatory layer in cancer. Furthermore, competing endogenous RNA (ceRNA) networks, in which lncRNAs, circRNAs, pseudogenes, and mRNAs compete for shared miRNAs, are now recognized as key modulators of oncogenic signaling, cell cycle control, immune evasion, and therapy resistance [36,37]. In HCC, several ceRNA axes have been shown to regulate immune checkpoint expression, tumor metabolism, and metastatic potential [35,38]. These RNA-based regulatory circuits operate in close interplay with epigenetic mechanisms, because DNA methylation and chromatin remodeling influence miRNA expression and target accessibility [35,39]. Despite this recognition, the integration of transcriptional regulators, such as ZBTB40, into ceRNA networks and how these multi-layered interactions shape immune and tumor phenotypes in HCC remain largely unexplored [38].

Therefore, we aimed to perform a comprehensive multi-omics and bioinformatics analysis to elucidate the expression patterns, prognostic significance, epigenetic regulation, immune associations, and miRNA network of ZBTB40 in HCC.

## 2. Materials and Methods

### 2.1. Data Collection and Processing

Publicly available transcriptomic and clinical data for HCC were retrieved from TCGA-HCC resources. Because the present study used de-identified open-access datasets, additional institutional review board approval or patient consent was not required. Samples were included only when the variables required for each analysis were available. Cases lacking essential gene expression values, survival information, or relevant clinicopathological annotations were excluded from the corresponding analyses. After filtering, 374 tumor samples and 50 non-tumor liver samples were used for expression-based comparisons. Expression values were processed according to the normalization pipeline of each database or analytical platform and were log2-transformed when required.

### 2.2. Analysis of ZBTB40 Expression

The expression profile of ZBTB40 was examined in HCC and non-tumor liver tissues using TIMER3.0 (http://timer.cistrome.org/; accessed on 7 August 2025), Wanderer (http://maplab.cat/wanderer; accessed on 7 August 2025), and UALCAN (http://ualcan.path.uab.edu; accessed on 8 August 2025). These platforms were applied as complementary resources to verify ZBTB40 expression patterns across publicly available cancer transcriptomic datasets [40,41,42]. Tumor–normal comparisons were first performed, and subgroup analyses were then conducted according to available clinicopathological variables, including tumor stage, tumor grade, sex, age, race, histological subtype, and TP53 mutation status [40].

### 2.3. Survival Analysis

The relationship between ZBTB40 expression and patient survival was evaluated using Kaplan–Meier Plotter (http://kmplot.com/analysis/; accessed on 10 August 2025), GEPIA2 (http://gepia2.cancer-pku.cn/; accessed on 10 August 2025), and GSCA (https://guolab.wchscu.cn/GSCA/; accessed on 11 August 2025) [38,43,44]. Patients were classified into high- and low-expression groups according to the median or platform-defined cutoff value for ZBTB40 expression. Overall survival (OS), progression-free survival (PFS), disease-specific survival (DSS), and disease-free interval (DFI) were analyzed when the corresponding endpoints were available. Survival differences were assessed using log-rank tests, and hazard ratios (HRs) with 95% confidence intervals were obtained from each platform.

### 2.4. Immune Infiltration Analysis

The association between ZBTB40 expression and immune infiltration was investigated using TIMER3.0 and GSCA. Estimated immune cell (including B cells, CD4+ and CD8+ T cells, macrophages, neutrophils, and dendritic cells) abundance values were correlated with ZBTB40 expression across tumor samples. Associations with immune cell subsets, immune-related genes, immune subtypes, and immune checkpoint molecules were assessed using Spearman correlation-based analyses.

### 2.5. DNA Methylation Analysis

ZBTB40 DNA methylation patterns were assessed using UALCAN, SMART (http://smart.embl.de; accessed on 18 September 2025), OncoDB (https://oncodb.org/; accessed on 22 September 2025), MEXPRESS (https://mexpress.be/; accessed on 28 September 2025), and MethSurv (https://biit.cs.ut.ee/methsurv; accessed on 29 September 2025). Promoter methylation levels were compared between HCC and non-tumor liver tissues and were further examined across available clinical subgroups [40]. CpG-level methylation patterns and expression–methylation relationships were visualized using SMART and MEXPRESS. MethSurv was used to examine the survival relevance of selected ZBTB40-associated CpG probes. Probes with missing methylation values were excluded from downstream interpretation [45].

### 2.6. Co-Expression and Functional Enrichment Analysis

ZBTB40-associated transcriptomic patterns were analyzed using LinkedOmics (http://www.linkedomics.org; accessed on 15 April 2025). Genes were ranked according to Pearson correlation coefficients with ZBTB40 expression in HCC. Functional enrichment analysis was performed through the LinkInterpreter module to identify Gene Ontology terms and Kyoto Encyclopedia of Genes and Genomes pathways associated with ZBTB40-correlated genes. Enrichment significance was interpreted using the default LinkedOmics workflow with false discovery rate correction.

### 2.7. Construction of ceRNA Network

Candidate miRNAs predicted to interact with ZBTB40 were identified using miRNet 2.0 (https://www.mirnet.ca; accessed on 15 August 2025) and ENCORI (starBase v3.0) (https://starbase.sysu.edu.cn; accessed on 16 August 2025) [45,46,47]. Upstream non-coding RNA candidates, including lncRNAs, circRNAs, pseudogenes, and small non-coding RNAs, were then retrieved from these network resources. The survival relevance of selected miRNAs and non-coding RNA candidates was assessed using Kaplan–Meier survival outputs available in ENCORI.

### 2.8. Gene–Chemical Interaction Analysis

ZBTB40-related chemical–gene interaction records were obtained from CTD (CTD; https://ctdbase.org/; accessed on 20 September 2025) and GSCA. Chemicals were categorized according to whether they were associated with increased or decreased ZBTB40 expression [44,48]. A similarity-based analysis was subsequently performed to identify genes sharing overlapping chemical interaction profiles with ZBTB40. This analysis was used to provide a toxicogenomic overview of ZBTB40-associated chemical signatures.

### 2.9. External Cohort Validation

External validation was performed using the HCCDB6/GSE14520 cohort. ZBTB40 expression was compared between HCC and adjacent non-tumor liver tissues. Survival analysis was additionally conducted according to ZBTB40 expression status when survival information was available.

### 2.10. Network Analysis and Multivariable Cox Regression

Protein–protein interaction (PPI) networks were generated using STRING v10.0 (https://version10.string-db.org/; accessed on 14 September 2025) [49] and GeneMANIA (https://genemania.org/; accessed on 16 September 2025) [50,51]. Physical interactions, co-expression, co-localization, shared protein domains, and pathway-based relationships were considered when constructing the ZBTB40-associated interaction network. To determine whether the prognostic association of ZBTB40 was independent of available clinical variables, multivariable Cox proportional hazards regression analysis was performed using the TCGA-LIHC cohort. ZBTB40 expression, age, gender, and tumor stage were included in the model. Hazard ratios and 95% confidence intervals were calculated.

### 2.11. Statistical Analysis

Statistical analyses were performed using the analytical functions embedded in each public platform. Continuous variables were compared using Student’s t-test or Wilcoxon rank-sum test, depending on the data structure and platform-specific settings. Pearson or Spearman correlation coefficients were used for association analyses between continuous variables. Survival outcomes were evaluated using log-rank tests and Cox proportional hazards models. False discovery rate correction was applied when available. A *p*-value < 0.05 was considered statistically significant.

## 3. Results

### 3.1. ZBTB40 Is Upregulated in HCC and Associated with Clinicopathological Features

**Figure 1:**
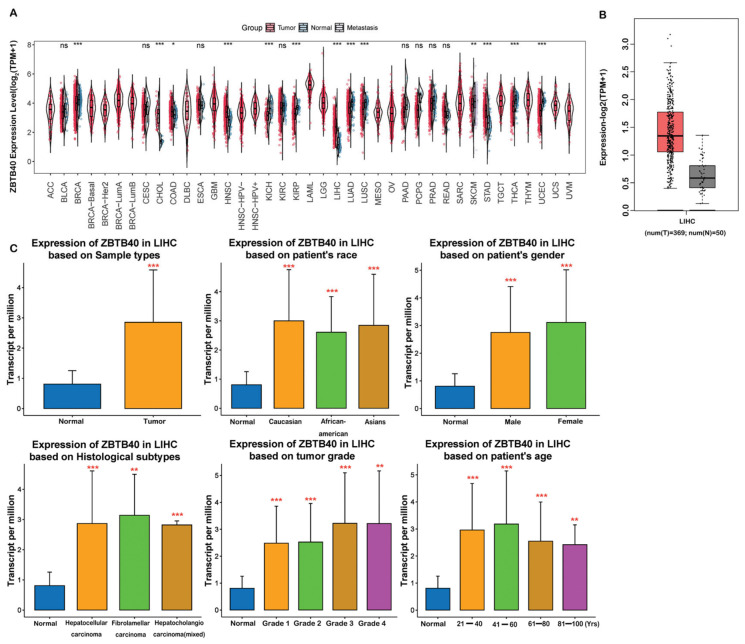
mRNA expression pattern of ZBTB40 in HCC and pan-cancer datasets. (**A**) Pan-cancer analysis of ZBTB40 expression across tumor, normal, and metastatic tissues. Red indicates tumor tissues, blue indicates normal tissues, and gray indicates metastatic tissues. (**B**) ZBTB40 expression in LIHC tumor and normal tissues. (**C**) ZBTB40 expression in LIHC according to sample type, race, gender, histological subtype, tumor grade, and age. Statistical significance is indicated as * *p* < 0.05, ** *p* < 0.01, and *** *p* < 0.001; ns, not significant.

ZBTB40 mRNA expression was markedly elevated across multiple cancer types compared with corresponding normal tissues, with significant upregulation observed in HCC and several other malignancies (Figure 1A). In HCC, independent comparison of tumor and normal tissues further confirmed that ZBTB40 expression was significantly higher in tumor tissues than in normal liver tissues (Figure 1B). To further characterize the clinical relevance of ZBTB40 in HCC, its expression was analyzed across multiple clinicopathological subgroups. ZBTB40 expression was significantly increased in primary tumor tissues and remained elevated across diverse clinical subgroups, including patient race, gender, histological subtype, tumor grade, and age groups, when compared with normal tissues (Figure 1C). Collectively, these findings indicate that ZBTB40 is aberrantly overexpressed in HCC and is associated with multiple clinicopathological characteristics, suggesting its potential clinical relevance in HCC.

### 3.2. Prognostic Relevance of ZBTB40 Expression in HCC

**Figure 2:**
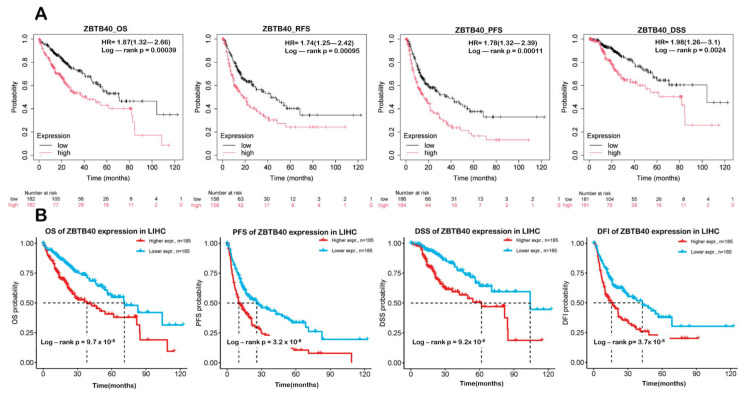
The prognostic significance of ZBTB40 gene expression in HCC. Kaplan–Meier survival analyses comparing high and low ZBTB40 expression groups in HCC. (**A**) Prognostic survival curves generated from the Kaplan–Meier Plotter database are shown. (**B**) Survival analyses based on the GSCA platform, demonstrating the prognostic value of ZBTB40 expression in HCC across multiple survival endpoints, including overall survival (OS), progression-free survival (PFS), disease-specific survival (DSS), and disease-free interval (DFI). Patients were stratified into high- and low-expression groups according to median expression levels. Hazard ratios (HRs), 95% confidence intervals, and log-rank *p*-values are indicated.

We examined whether ZBTB40 expression was associated with survival outcomes in HCC. Kaplan–Meier Plotter and GSCA analyses showed that patients with higher ZBTB40 expression generally had poorer survival outcomes than those with lower expression (Figure 2). Across the analyzed endpoints, elevated ZBTB40 expression was associated with unfavorable overall survival, relapse-free survival, progression-free survival, disease-specific survival, and disease-free interval (Figure 2A,B). Subgroup analyses further showed that the survival-related pattern of ZBTB40 expression was observed in several clinically relevant patient categories, including selected stage, grade, sex, ethnicity, alcohol consumption, vascular invasion, and hepatitis virus infection subgroups. Because the subgroup-specific hazard ratios were extensive, the detailed results are summarized in Table 1 rather than described individually in the main text.

External validation using the HCCDB6/GSE14520 cohort further supported the association between high ZBTB40 expression and unfavorable survival outcomes in HCC. These results are provided in Supplementary Figure S5. In addition, multivariable Cox proportional hazards regression analysis was performed using the TCGA-LIHC cohort. After adjustment for available clinicopathological variables, including age, gender, and tumor stage, high ZBTB40 expression remained significantly associated with poorer overall survival (HR = 1.682, 95% CI = 1.352–2.361, *p* < 0.001). These findings are summarized in Supplementray Table S5. Collectively, these results suggest that ZBTB40 expression is associated with unfavorable survival outcomes in HCC and may represent a candidate prognostic marker requiring further validation.

### 3.3. ZBTB40 Expression Is Closely Associated with Immune Infiltration Patterns in HCC

**Figure 3:**
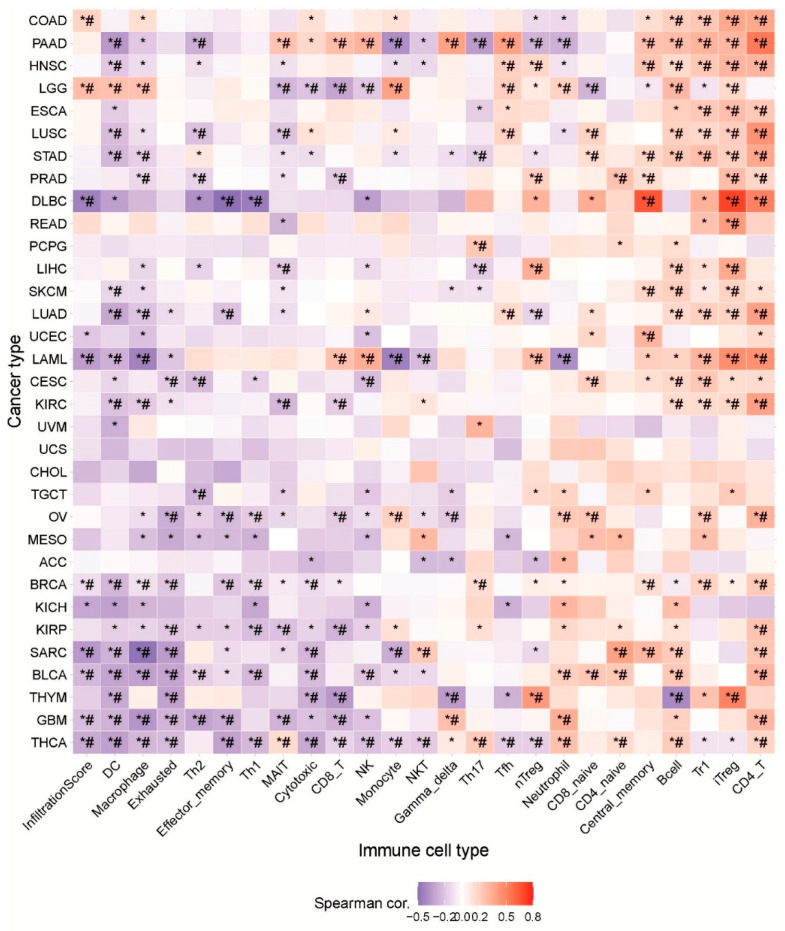
The immune infiltration landscape associated with ZBTB40 expression. Spearman’s correlation coefficients between ZBTB40 expression and immune cell infiltration levels across multiple cancer types. Rows represent cancer types, and columns indicate immune cell subsets. The color intensity reflects the strength and direction of the correlations (red, positive; purple, negative). Statistical significance is denoted by * (*p* < 0.05) and # (FDR < 0.05).

We analyzed the relationship between ZBTB40 expression and tumor-infiltrating immune cells across multiple cancer types using the GSCA platform to explore the immunological relevance of ZBTB40 (Figure 3). ZBTB40 expression exhibited heterogeneous immune association patterns across cancers, indicating that the immunological relevance of ZBTB40 is highly dependent on the tumor type. Among all cancer types, HCC showed one of the most distinct and statistically significant immune association profiles for ZBTB40 expression. In HCC, ZBTB40 expression was significantly correlated with multiple immune cell subsets, indicating that ZBTB40 is associated with the immune microenvironment in liver tumors. Specifically, ZBTB40 expression in HCC was significantly associated with cytotoxic immune populations, including CD8+ T and natural killer (NK) cells, suggesting that ZBTB40 expression may be associated with antitumor immune-related signatures. ZBTB40 expression was also significantly correlated with CD4+ T, T helper (Th), and effector memory T cells, indicating a broad association with adaptive immune responses. Notably, ZBTB40 expression was significantly correlated with immunosuppressive and inflammatory cell populations, including regulatory T cells (Tregs), macrophages, monocytes, and neutrophils. These immune subsets contribute to immune evasion, tumor-promoting inflammation, and resistance to immunotherapy in HCC. The simultaneous association of ZBTB40 with cytotoxic and suppressive immune compartments suggests that ZBTB40 expression is associated with a complex immune infiltration profile in HCC. Pan-cancer immune correlation heatmaps revealed that ZBTB40 expression was associated with immune infiltration patterns, with particular relevance in HCC. Many of these correlations remained statistically significant after FDR correction, supporting the robustness of the observed immune associations.

### 3.4. ZBTB40 Multi-Omics Features Show Distinct Associations with Immune Infiltration in HCC

**Figure 4:**
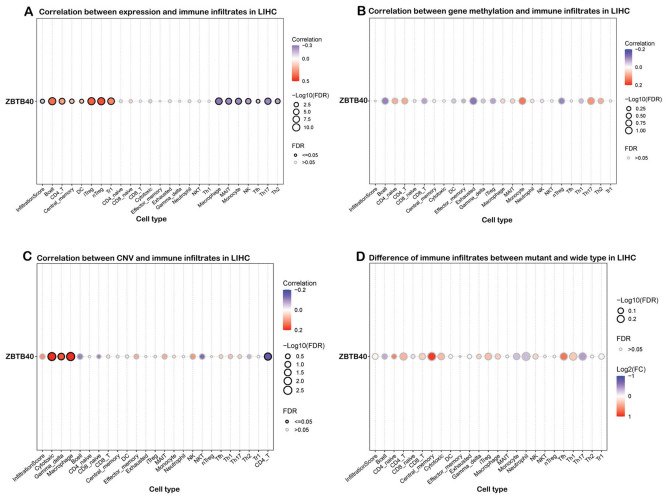
The associations between ZBTB40 multi-omics features and immune cell infiltration in HCC. (**A**) Correlation analysis between ZBTB40 mRNA expression and estimated immune cell infiltration across immune cell subsets in HCC. (**B**) Correlation analysis between ZBTB40 DNA methylation levels and immune cell infiltration in HCC. (**C**) Correlation analysis between ZBTB40 copy number variation (CNV) status and immune cell infiltration in HCC. (**D**) Differences in estimated immune cell infiltration between ZBTB40-mutant and wild-type HCC tumors. (**A**–**C**) Dot color indicates the direction and magnitude of the correlation (red, positive; blue, negative), and dot size represents −log10(FDR). (**D**) Dot color represents log2 fold change (mutant vs. wild-type) and dot size represents −log10(FDR). Statistical significance is indicated by an FDR ≤ 0.05.

We assessed the relationship between immune infiltration estimates and multiple molecular layers of ZBTB40, including mRNA expression, DNA methylation, copy number variation (CNV), and somatic mutation status, using the GSCA platform to investigate the immunological relevance of ZBTB40 in HCC (Figure 4). ZBTB40 mRNA expression displayed a broad immune association profile across immune subsets (Figure 4A). In particular, positive correlations (red dots) were observed between ZBTB40 and several immune compartments, including B, CD4+ T, and dendritic cells (DC), and regulatory T cell subsets such as iTregs and nTregs. Additionally, ZBTB40 expression was positively associated with central memory T cells and Tr1, suggesting that higher ZBTB40 expression is accompanied by increased immune infiltration signatures in these populations (Figure 4A). Conversely, significant negative correlations (blue/purple dots) were observed between ZBTB40 expression and multiple immune cell subsets, including Th1, macrophages, MAIT, monocytes, NK, Tfh, Th17, and Th2 cells (Figure 4A). Notably, the negative associations in the right-hand immune subsets were visually more frequently accompanied by larger dot sizes, indicating more robust FDR support (Figure 4A). Collectively, these findings suggest that ZBTB40 expression is linked to a complex immune infiltration pattern in HCC, characterized by the enrichment of certain lymphoid/regulatory components and depletion of multiple effector/innate immune subsets. In contrast, transcript-level analyses indicated weak correlations between ZBTB40 DNA methylation and immune infiltration, and no immune subset reached FDR ≤ 0.05 in this panel. Although modest trend-level correlations were visible for the selected subsets (mixed red/blue dots), they did not pass FDR correction based on the plot annotation (Figure 4B). Therefore, ZBTB40 methylation was not significantly associated with immune infiltration patterns in HCC in this dataset and analytical setting. We then examined whether CNV in ZBTB40 in tumor was linked to immune infiltration (Figure 4C). CNV status showed significant associations (FDR ≤ 0.05) with a limited set of immune subsets, rather than a broad pattern (Figure 4C). Specifically, significant positive associations were observed between CNV status and cytotoxic and gamma-delta T cells and macrophages (large red dots with FDR significance), suggesting that the ZBTB40 CNV may be related to increased infiltration or enrichment of these immune populations (Figure 4C). In contrast, a significant negative association was evident between CNV status and CD4+ T cells (blue dot with FDR significance), indicating that the ZBTB40 CNV is linked to reduced CD4+ T cell infiltration estimates (Figure 4C). Other immune subsets displayed smaller dots with an FDR > 0.05, indicating either weak effects or a lack of statistical robustness. Finally, we compared the immune cell infiltration levels between ZBTB40-mutant and wild-type HCC tumors. As shown in Figure 4D, differences across immune subsets were generally small and did not reach FDR ≤ 0.05 (the legend displays FDR > 0.05 only). Although several subsets showed directionally higher or lower infiltration in the mutant group (color-coded by log2 fold change), they did not demonstrate statistical robustness after correction (Figure 4D). Thus, the ZBTB40 mutation status alone did not appear to drive large-scale immune infiltration shifts in this cohort. The immune relevance of ZBTB40 in HCC was most pronounced at the transcript level (mRNA expression), with a more limited association observed at the CNV level. DNA methylation and somatic mutation status showed weak or non-significant associations with immune infiltration after FDR correction. These findings demonstrate that ZBTB40 expression and selected genomic features are associated with distinct immune infiltration patterns in HCC, warranting further investigation of their biological and clinical significance.

### 3.5. ZBTB40 Is Associated with DNA Methylation in HCC

**Figure 5:**
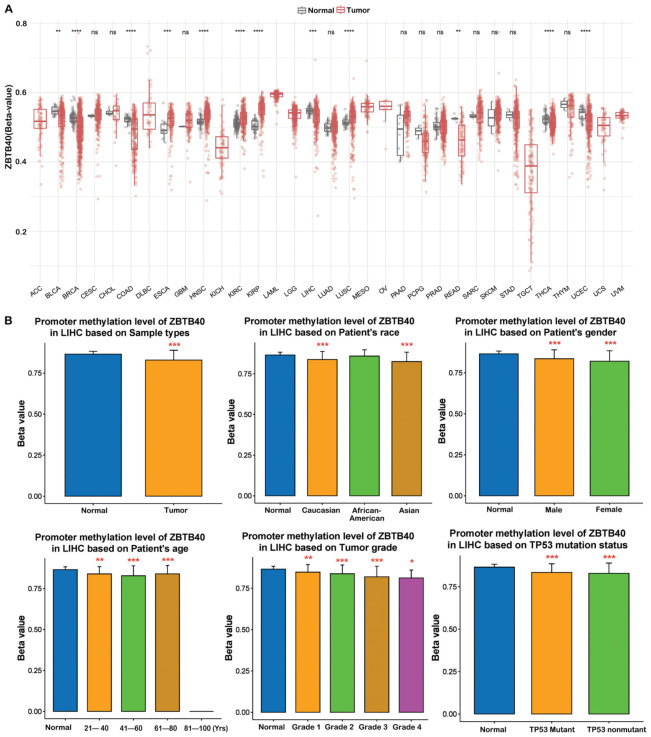
ZBTB40 DNA methylation patterns in HCC. (**A**) Comparison of ZBTB40 promoter DNA methylation levels between tumor and normal tissues across multiple cancer types. (**B**) Comparison of ZBTB40 promoter methylation levels in HCC according to sample type, patient race, sex, tumor grade, patient age, and TP53 mutation status. Statistical significance is indicated by * *p* < 0.05, ** *p* < 0.01, *** *p* < 0.001, and **** *p* < 0.0001.

**Figure 6:**
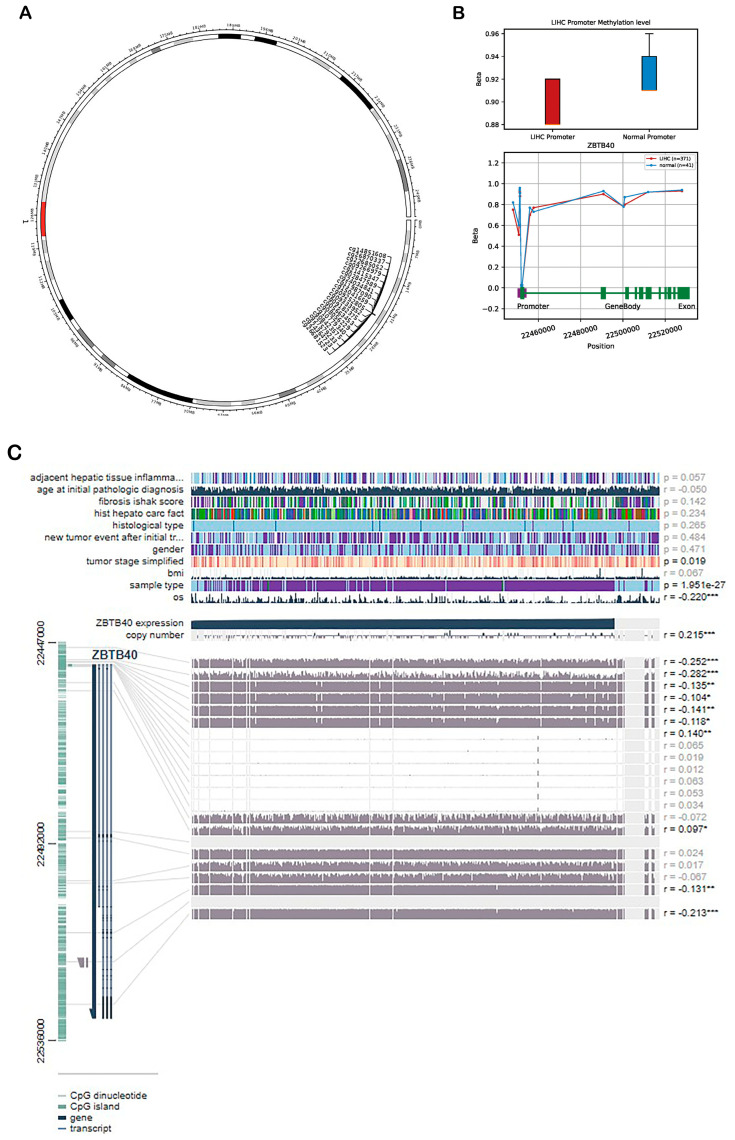
DNA methylation and probe-level correlation of ZBTB40 in HCC. (**A**) DNA methylation probes mapped to ZBTB40 in HCC. Gray segments indicate chromosomal ideograms, and colored regions show methylation-associated loci. (**B**) Comparison of ZBTB40 promoter DNA methylation levels between HCC tumor tissues and normal liver tissues. (**C**) Correlation of ZBTB40-associated probes in HCC. Statistical significance is indicated as * *p* < 0.05, ** *p* < 0.01, and *** *p* < 0.001.

To investigate the potential epigenetic relevance of ZBTB40, we analyzed its promoter methylation status across multiple cancer types and evaluated its clinicopathological associations in HCC. Cancer analysis revealed that ZBTB40 promoter methylation levels differed significantly between tumor and normal tissues in several malignancies (Figure 5A). In HCC, tumor samples exhibited significantly lower beta values than normal liver tissues, indicating promoter hypomethylation in cancer tissues. Similar differential methylation patterns were observed in multiple cancer types, although the magnitude and direction of changes varied among tumors. The distribution of beta values demonstrated a consistent shift toward reduced methylation levels in HCC tumors, suggesting epigenetic deregulation of the ZBTB40 locus in HCC (Figure 5A). Subgroup analyses were conducted based on the clinicopathological characteristics to further delineate the clinical relevance of ZBTB40 methylation in HCC (Figure 5B). When stratified by sample type, HCC tumor tissues showed significantly decreased promoter methylation compared to normal tissues, confirming tumor-associated hypomethylation (Figure 5B, sample types). Stratification by race revealed that methylation levels remained significantly reduced across Caucasian, African-American, and Asian patient groups compared to normal controls, indicating that the hypomethylation pattern was not restricted to a specific ethnic background (Figure 5B). Gender-based analysis revealed that male and female patients with HCC exhibited significantly lower promoter methylation levels than normal liver tissues, suggesting that ZBTB40 hypomethylation occurs independent of sex (Figure 5B, patient sex). Age-stratified analysis further demonstrated that reduced methylation was consistent across different age groups (21–40, 41–60, and 61–80 years), indicating that the epigenetic alteration of ZBTB40 is maintained throughout the adult age ranges in HCC (Figure 5B, patient age). When evaluated according to tumor grade, decreased promoter methylation was observed across grades 1–4, with a trend toward persistent hypomethylation, regardless of the differentiation status, suggesting that this epigenetic change is present throughout tumor progression (Figure 5B, Tumor grade). Furthermore, a comparison based on TP53 mutation status showed that TP53-mutant and TP53-nonmutant tumors exhibited significantly lower methylation levels than normal tissues, indicating that ZBTB40 hypomethylation is not confined to a specific TP53 genomic background (Figure 5B, TP53 mutation status). To assess the potential epigenetic regulation of ZBTB40 in HCC, its chromosomal location and methylation profile were examined (Figure 6). ZBTB40 was located on chromosome 1 (Figure 6A). Comparative analysis of promoter methylation showed a tendency toward lower methylation levels in HCC tissues than in normal liver tissues (Figure 6B). Correlation analysis demonstrated that methylation at these ZBTB40-associated probes was negatively correlated with methylation levels at several CpG sites, including cg24834590 (R = –0.252, *p* < 0.001), cg14851608 (R = –0.282, *p* < 0.001), cg26870337 (R = –0.135, *p* < 0.01), cg22685052 (R = –0.104, *p* < 0.05), cg09266979 (R = –0.141, *p* < 0.01), cg24364723 (R = –0.131, *p* < 0.01), and cg26681523 (R = –0.213, *p* < 0.001). Conversely, several probes exhibited positive correlations with ZBTB40 expression, including cg02847589 (R = 0.140, *p* < 0.01) and cg08056229 (R = 0.097, *p* < 0.05) (Figure 6C). These findings suggest an association between promoter methylation status and ZBTB40 expression in HCC.

### 3.6. DNA Methylation Patterns of ZBTB40 in HCC

**Figure 7:**
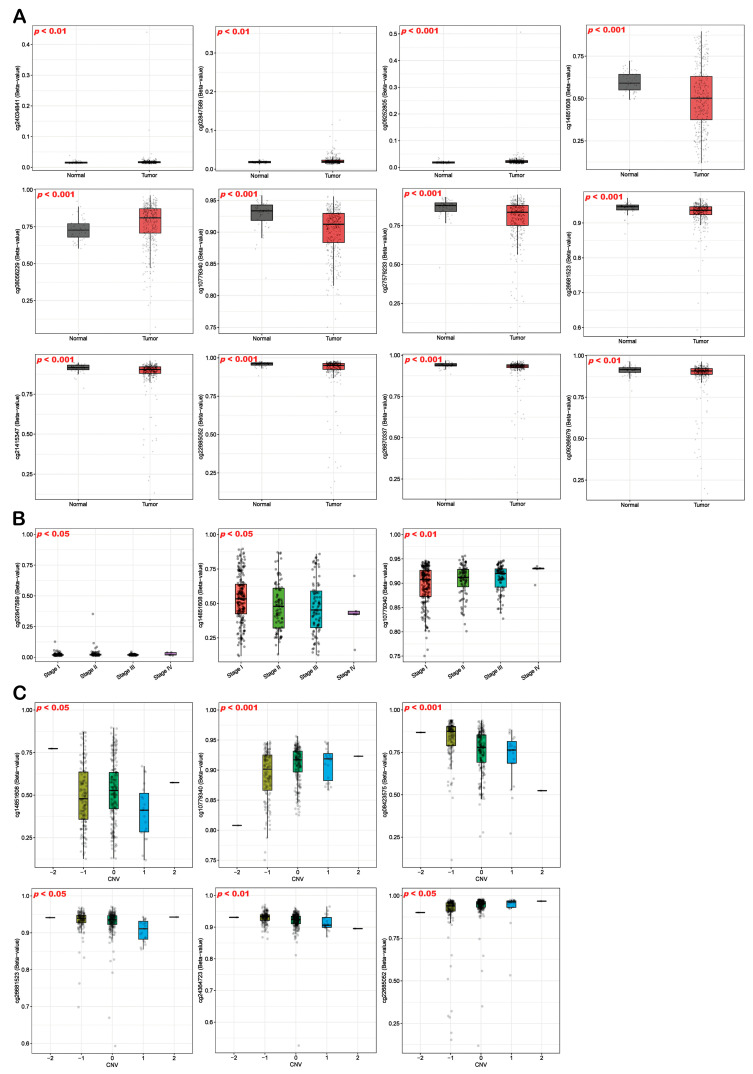
Chemical–gene interaction analysis of ZBTB40 in HCC. (**A**) Chemicals associated with increased ZBTB40 expression. (**B**) Chemicals associated with decreased ZBTB40 expression. (**C**) Network visualization of ZBTB40-associated gene–chemical interactions. In panels A and B, green bars indicate chemicals associated with increased ZBTB40 expression, whereas red bars indicate chemicals associated with decreased ZBTB40 expression. In panel C, nodes represent genes and chemicals, and lines indicate reported gene–chemical interactions. Different node colors distinguish ZBTB40, ZBTB40-associated genes, and interacting chemical compounds.

We analyzed differential DNA methylation levels at multiple CpG sites in normal liver and tumor tissues to characterize the epigenetic landscape of ZBTB40 in HCC. A comparison of the beta values representing methylation levels is indicated in each panel (Figure 7A). Among the analyzed CpG loci, cg02847589 demonstrated a modest but statistically significant increase in methylation in tumors compared to that in normal tissues (*p* < 0.01), with a greater dispersion observed in the tumor group. In contrast, cg08056229 showed markedly elevated methylation levels in tumor tissues compared to that in normal samples (*p* < 0.001), accompanied by substantial inter-tumoral heterogeneity. Similarly, cg09252805 exhibited significantly higher methylation in tumors (*p* < 0.001), although the absolute beta values remained within a relatively low methylation range. Notably, cg09266979 displayed a minimal but significant reduction in methylation in tumor tissues (*p* < 0.01), whereas cg10779340 and cg22685052 demonstrated significantly decreased methylation levels in tumors compared to normal tissues (both *p* < 0.001), indicating potential site-specific hypomethylation events. Additionally, cg14851608 showed pronounced hypomethylation in tumor tissues (*p* < 0.001) with a wide distribution of beta values, suggesting substantial epigenetic variability among HCC samples. Conversely, CpG sites such as cg21415347, cg26681523, and cg26870337 maintained relatively high methylation levels in normal and tumor tissues but exhibited statistically significant reductions in tumors (all *p* < 0.001), reflecting subtle yet consistent epigenetic alterations. Furthermore, cg24034841 showed a slight increase in tumor methylation levels (*p* < 0.01), whereas cg27579233 showed a clear decrease in tumor methylation compared to normal tissues (*p* < 0.001). As a result, the methylation levels at multiple CpG loci varied significantly across the CNV states. At cg08423575, methylation significantly differed across CNV categories (−2, −1, 0, 1, 2) (*p* < 0.001), demonstrating higher β-values in shallow deletion and diploid states compared with copy number gain groups (Figure 7B). Similarly, cg10779340 exhibited significant methylation variation across the CNV strata (*p* < 0.001), with altered distribution patterns among the deletion, neutral, and amplification states. Additional CpG sites showed consistent CNV-dependent differences in methylation. At cg14851608 and cg22685052, the methylation levels varied significantly across the CNV categories (*p* < 0.05), indicating that genomic dosage changes were associated with altered epigenetic profiles. Similarly, cg24364723 (*p* < 0.01) and cg26681523 (*p* < 0.05) demonstrated significant methylation shifts according to CNV status, further supporting the relationship between structural genomic variation and DNA methylation dynamics.

Stage-specific methylation patterns were then analyzed using TCGA-HCC data to determine whether ZBTB40 methylation levels are associated with tumor progression. The beta values for the selected CpG probes were compared across pathological stages I–IV. The methylation levels of the probe cg02847589 exhibited statistically significant differences across tumor stages (*p* < 0.05). Similarly, cg10779340 showed significant age-related methylation differences (*p* < 0.01). For cg14851608, methylation levels also differed significantly among the clinical stages (*p* < 0.05) (Figure 7C). Stage I samples showed broader dispersion, whereas stage II and III exhibited comparatively shifted median values, indicating potential remodeling of DNA methylation during disease progression. Collectively, these analyses demonstrate that ZBTB40 DNA methylation is significantly associated with both genomic copy number alterations and clinicopathological stages of HCC. Coordinated variation across structural, epigenetic, and clinical parameters supports a stage- and CNV-dependent epigenetic remodeling pattern at the ZBTB40 locus.

### 3.7. Integrated Analysis of ZBTB40-Associated miRNAs and ceRNA Networks in HCC

**Figure 8:**
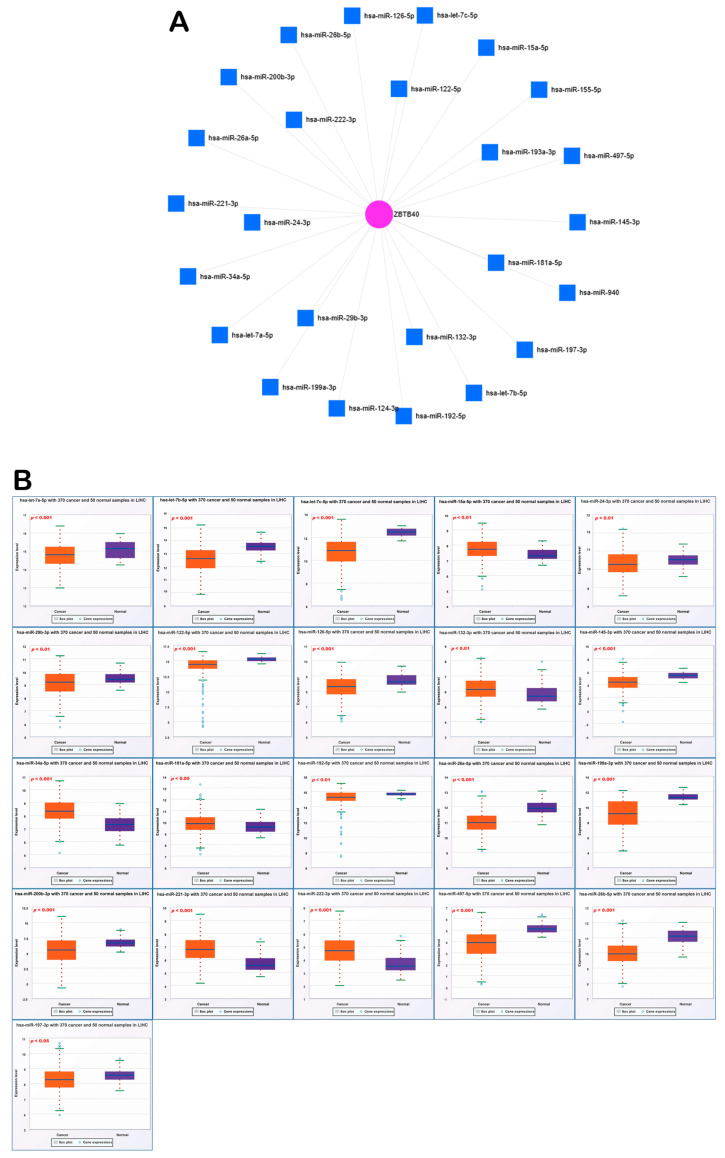

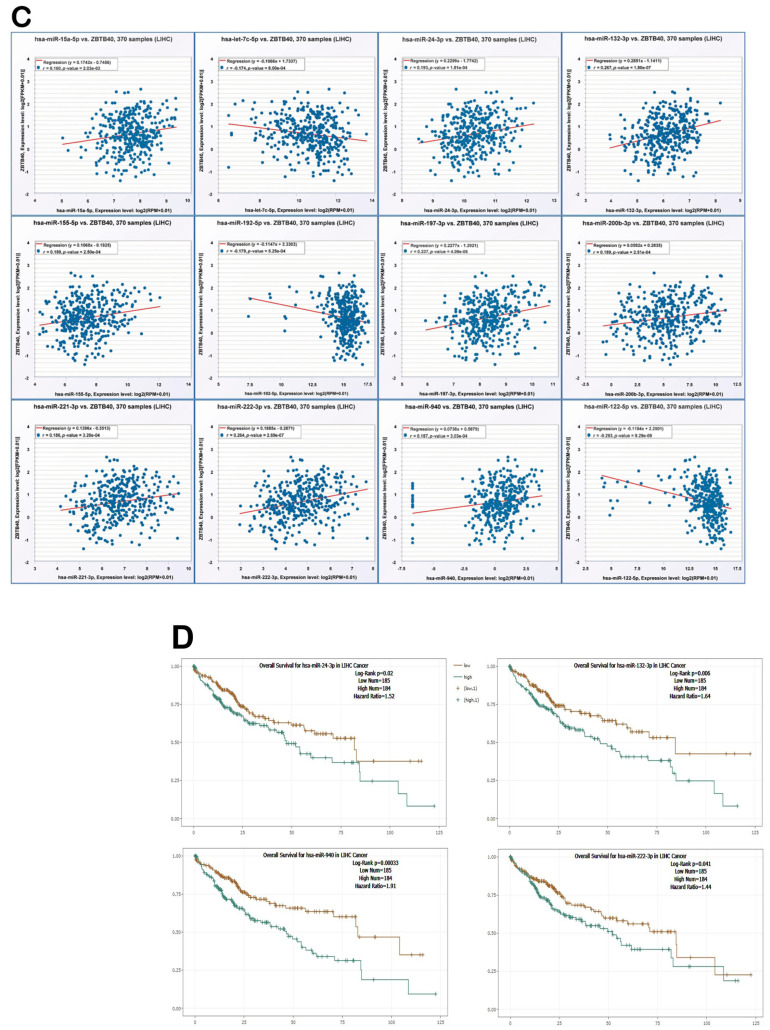
ZBTB40-associated miRNAs in HCC. (**A**) miRNA network associated with ZBTB40. (**B**) Expression of ZBTB40-associated miRNAs in HCC. (**C**) Correlation between ZBTB40 and ZBTB40-associated miRNAs in HCC. (**D**) KM plotter of ZBTB40-associated miRNAs. *p*-values were calculated using the log-rank test, and hazard ratios are shown in each plot.

To characterize post-transcriptional regulatory features associated with ZBTB40 in HCC, we first screened candidate miRNAs predicted to interact with ZBTB40 using miRNet. This analysis generated a ZBTB40-centered miRNA interaction map containing 41 candidate miRNAs (Figure 8A). We then evaluated the expression patterns of these candidate miRNAs between HCC and normal liver tissues. Several predicted miRNAs showed tumor-associated downregulation, whereas others were upregulated in HCC, indicating heterogeneous expression patterns among ZBTB40-related miRNAs (Figure 8B).

Correlation analysis using TCGA-HCC samples further revealed distinct relationships between ZBTB40 expression and selected candidate miRNAs. hsa-miR-122-5p, hsa-miR-192-5p, and hsa-let-7c-5p showed inverse correlations with ZBTB40 expression, whereas hsa-miR-132-3p, hsa-miR-222-3p, hsa-miR-197-3p, hsa-miR-24-3p, and hsa-miR-155-5p showed positive correlations (Figure 8C). Survival analysis additionally showed that higher expression of hsa-miR-24-3p, hsa-miR-132-3p, hsa-miR-222-3p, and hsa-miR-940 was associated with poorer overall survival in HCC (Figure 8D).

Based on differential expression, correlation with ZBTB40, and survival relevance, hsa-miR-24-3p, hsa-miR-132-3p, hsa-miR-222-3p, and hsa-miR-940 were selected for downstream ceRNA network analysis. Predicted interaction maps linked these miRNAs to multiple classes of non-coding RNAs, including lncRNAs, circRNAs, pseudogenes, and small non-coding RNAs. CircRNAs accounted for the largest proportion of predicted interacting molecules across the analyzed networks. Representative miRNA-level findings are summarized in Figure 8, whereas complete ceRNA interaction maps and candidate RNA lists are provided in Supplementary Figures S1–S4 and Supplementary Tables S1–S4.

Differential expression analyses showed that several candidate lncRNAs and circRNA-associated genes were dysregulated in HCC tissues compared with normal liver tissues. Notably, lncRNAs such as CYTOR, DUXAP8, SNHG16, TUG1, VASH1-AS1, LINC00662, and MIR4435-2HG were elevated in tumor tissues. In addition, multiple circRNA-associated genes showed increased expression in HCC. Survival analyses further indicated that several components of the predicted ceRNA networks were associated with overall survival, with higher expression generally corresponding to unfavorable clinical outcomes.

Collectively, these findings suggest that ZBTB40-associated miRNAs are embedded within putative ceRNA networks involving diverse coding and non-coding RNA species. Although these relationships are based on computational predictions and require experimental validation, the observed expression, correlation, and survival patterns suggest potential relevance of ZBTB40-related ceRNA networks in HCC. Detailed network structures, candidate molecule lists, and individual survival analyses are provided in the Supplementary Materials.

### 3.8. Co-Expression and Functional Enrichment Analysis of ZBTB40 in HCC

**Figure 9:**
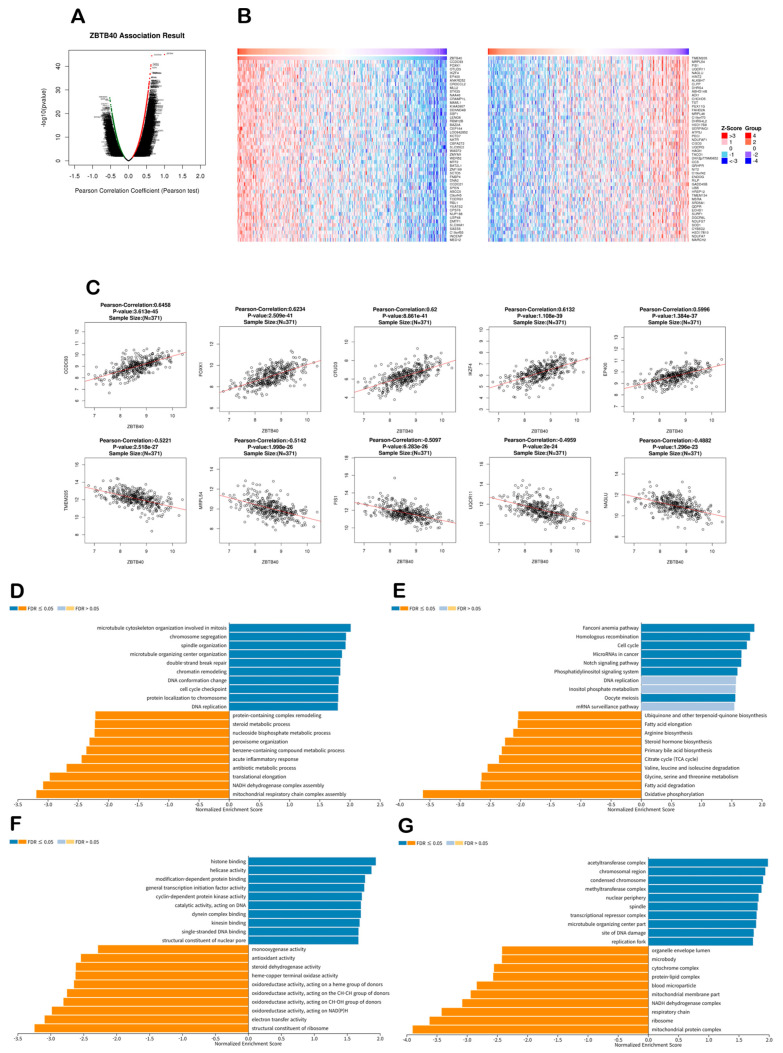
Co-expression and functional enrichment analysis of ZBTB40 expression in HCC. (**A**) Volcano plot showing genes positively (dark red dots) and negatively (dark green dots) correlated with ZBTB40 expression in HCC. Heatmaps displaying top 50 genes (**B**) positively and (**C**) negatively correlated with ZBTB40 expression. (**D**) Correlation plots of top five genes positively and negatively correlated with ZBTB40 expression (line represents regression line). Gene Set Enrichment Analysis (GSEA) evaluating enriched (**E**) Gene Ontology (GO) biological processes, (**F**) GO cellular components, (**G**) GO molecular functions. Dark blue and orange bars indicate false discovery rate (FDR) ≤ 0.05.

**Figure 10:**
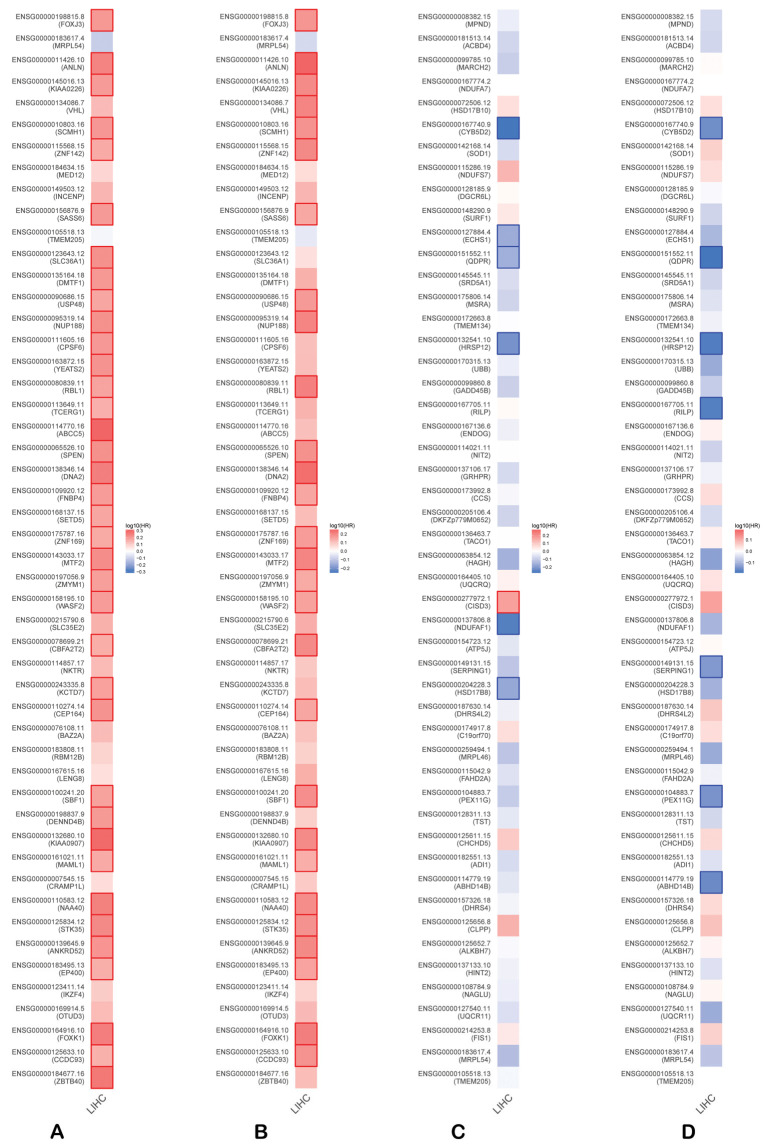
The prognostic significance of ZBTB40-related genes in HCC. Survival maps of genes positively correlated with ZBTB40 expression for OS (**A**) and RFS (**B**). Survival maps of genes negatively correlated with ZBTB40 expression for OS (**C**) and RFS (**D**). The heatmaps show log10(HR) values for each gene in HCC. Red indicates higher log10(HR) values, whereas blue indicates lower log10(HR) values. Squares with bold borders indicate statistically significant survival associations (*p* < 0.05).

A comprehensive transcriptome-wide correlation analysis identified ZBTB40 as a central node linked to extensive alterations in HCC gene expression (Figure 9A). Additionally, Pearson correlation-based analysis identified numerous transcripts that were positively or negatively correlated with ZBTB40 expression. Heatmap visualization of representative ZBTB40-associated genes revealed coordinated expression patterns across tumor samples (Figure 9B). Samples arranged along the ZBTB40 expression gradient displayed consistent upregulation of positively correlated genes and reciprocal downregulation of negatively correlated genes, suggesting a structured transcriptional program linked to ZBTB40 levels. Representative scatter plots further illustrated the strong linear relationships between ZBTB40 and key genes (Figure 9C). Notably, ZBTB40 showed strong positive correlations with CCDC83 (r = 0.6458, *p* = 3.613 × 10^−42^), FOXK1 (r = 0.6234, *p* = 2.509 × 10^−41^), OTUD3 (r = 0.6200, *p* = 8.861 × 10^−41^), KLF4 (r = 0.6132, *p* = 1.108 × 10^−39^), and EP400 (r = 0.5996, *p* = 1.384 × 10^−37^), all of which are functionally linked to transcriptional regulation, chromatin remodeling, or proliferative control. In contrast, ZBTB40 expression was inversely correlated with TMEM205 (r = −0.5221), MRPL54 (r = −0.5142), FIS1 (r = −0.5097), UQCR11 (r = −0.4959), and MAGLU (r = −0.4882) (all *p* ≤ 10^−23^), genes associated with mitochondrial structure, respiratory chain components, or metabolic regulation. These opposing correlation patterns suggest that ZBTB40 expression may delineate a shift between the proliferative and metabolic cellular states in HCC. Gene Set Enrichment Analysis (GSEA) provided a functional context for these associations. GO biological process enrichment (Figure 9D) demonstrated significant positive enrichment for mitotic microtubule organization, chromosome segregation, spindle assembly, DNA replication, double-strand break repair, chromatin remodeling, and cell cycle checkpoint regulation. KEGG pathway analysis (Figure 9E) further supported the enrichment of Fanconi anemia, homologous recombination, cell cycle, DNA replication, and Notch signaling pathways, collectively indicating the activation of genome stability and proliferative programs in tumors with higher ZBTB40 expression. Conversely, the negatively enriched pathways and functional categories were predominantly related to oxidative phosphorylation, fatty acid metabolism, steroid biosynthesis, oxidative stress-related oxidoreductase activity, electron transfer activity, ribosomal components, and mitochondrial respiratory chain complexes (Figure 9E–G). GO molecular function and cellular component analyses (Figure 9F,G) further highlighted the enrichment of chromosomal regions, replication forks, methyltransferase complexes, and transcriptional regulatory complexes on the positive side, and the suppression of mitochondrial membrane structures, NADH dehydrogenase complexes, cytochrome complexes, and ribosomal machinery on the negative side. Collectively, these results indicate that elevated ZBTB40 expression in HCC is associated with the transcriptional activation of cell cycle progression and DNA damage repair pathways, accompanied by the relative suppression of mitochondrial oxidative metabolism and translational machinery signatures. This transcriptional polarization suggests that ZBTB40 expression is associated with proliferative and metabolic reprogramming-related signatures in HCC. We analyzed survival data using the GEPIA2 database to evaluate the prognostic significance of ZBTB40-associated genes in HCC. The results indicated that genes positively correlated with ZBTB40 predominantly function as high-risk factors for HCC. A substantial number of positively associated genes exhibited elevated HRs for OS (Figure 10A), and a comparable proportion demonstrated high HR values for RFS (Figure 10B), indicating consistent associations with adverse clinical outcomes. In contrast, multiple negatively associated genes were associated with reduced HRs for OS (Figure 10C), and exhibited similar protective trends for RFS (Figure 10D). Collectively, these findings suggest that ZBTB40 expression and its positively correlated transcriptional modules may be associated with unfavorable clinical outcomes in HCC.

### 3.9. Toxicogenomic Associations of ZBTB40 Expression in HCC

We examined chemical–gene interaction records to explore whether ZBTB40 expression was linked to toxicogenomic signatures in HCC. Using CTD-derived interaction data, we identified chemical compounds associated with altered ZBTB40 expression (Table 2). Chemicals associated with increased ZBTB40 expression included several environmental toxicants and pharmacological agents, such as acrylamide, aflatoxin B1, aristolochic acid I, cadmium chloride, carbon tetrachloride, copper sulfate, gentamicins, sodium arsenite, and sorafenib. In contrast, decreased ZBTB40 expression was associated with compounds such as amphetamine, atrazine, bisphenol A, dietary fats, doxorubicin, folic acid, particulate matter, tetrachlorodibenzodioxin, thioacetamide, tretinoin, and vorinostat. To further characterize the chemical interaction profile of ZBTB40, a similarity index analysis was performed to identify genes with common interacting chemicals with ZBTB40 based on CTD (Table 3). Genes such as SAP30BP, TNK2, ADAMTSL5, PPP1R16A, SMYD4, CAMSAP1, TPRKB, RAB43, ZHX3, RNF13, CCNY, SFXN5, KCTD20, UBR3, NT5DC3, KDM2A, CHD3, IFT122, PARP6, NOP14, and TMEM214 showed high similarity indices with ZBTB40. Among these genes, RNF13 and CHD3 shared the largest number of ZBTB40-associated chemicals, followed by TNK2 and ZHX3. These findings suggest that ZBTB40 may be linked to a broader toxicogenomic interaction profile in HCC; however, these database-derived associations should be interpreted as hypothesis-generating.

### 3.10. Network-Based Identification and Co-Expression Validation of ZBTB40-Associated Hub Genes in HCC

**Figure 11:**
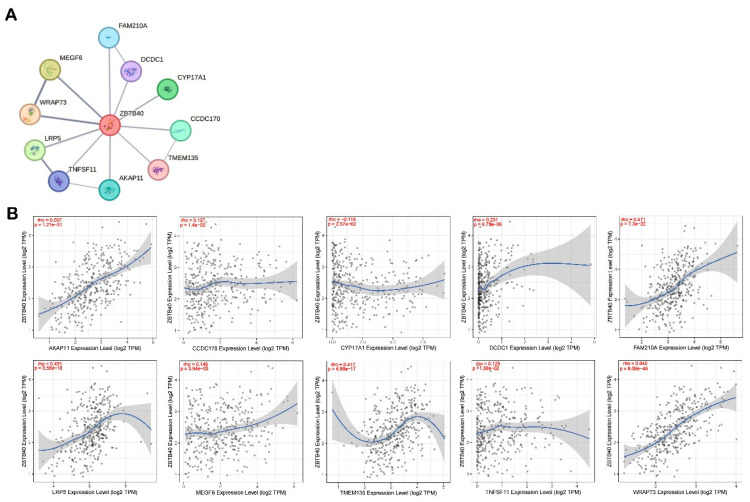
ZBTB40-centered interaction network and expression correlations in HCC. (**A**) A ZBTB40-centered protein–protein interaction (PPI) network highlighting the top candidate genes. (**B**) Co-expression analyses between ZBTB40 and each network partner in HCC using scatter plots of log2 TPM expression. Each dot represents an individual sample, the blue curve indicates the fitted trend, and the shaded area denotes the confidence interval. Correlation coefficients (ρ) and *p*-values are shown in each panel.

We analyzed the survival data using the GEPIA2 platform to evaluate the prognostic relevance of ZBTB40-associated genes in HCC (Figure 11). The analysis demonstrated that genes positively correlated with ZBTB40 expression exhibited unfavorable prognostic implications. Among these positively correlated genes, a substantial proportion were associated with increased HRs for OS and DFS, indicating their potential role as high-risk factors for HCC. In contrast, genes negatively correlated with ZBTB40 tended to display a comparatively favorable prognostic profile, with several candidates associated with reduced HRs for OS and DFS. Subsequently, pan-cancer heatmap analyses were performed to visualize the HR distributions across multiple tumor types, including HCC, to further illustrate the broader clinical impact of ZBTB40-associated gene sets. The positive association was observed for WRAP73 (ρ = 0.645, *p* = 6.09 × 10^−45^), followed by AKAP11 (ρ = 0.557, *p* = 1.21 × 10^−31^). Several genes showed moderate positive correlations, including FAM210A (ρ = 0.471, *p* = 7.3 × 10^−22^), LRP5 (ρ = 0.431, *p* = 3.56 × 10^−18^), and TMEM135 (ρ = 0.417, *p* = 4.98 × 10^−17^), indicating consistent co-variation with ZBTB40 across samples. DCDC1 expression also demonstrated a significant but weak positive correlation (ρ = 0.231, *p* = 6.79 × 10^−6^). In contrast, CCDC170 (ρ = 0.127, *p* = 1.4 × 10^−2^) and MEGF6 (ρ = 0.149, *p* = 3.94 × 10^−3^) displayed weak positive correlations, suggesting more modest coupling with ZBTB40 expression (Figure 11B). Notably, two candidates showed significant negative correlations with ZBTB40: CYP17A1 (ρ = −0.116, *p* = 2.57 × 10^−2^) and TNFSF11 (ρ = −0.125, *p* = 1.58 × 10^−2^) (Figure 11B). These analyses consistently highlighted the enrichment of high HR signals within the ZBTB40 positively correlated gene set, whereas the negatively correlated genes were more frequently associated with protective survival patterns. Collectively, these findings indicate that ZBTB40 and its positively co-expressed molecular network are closely associated with poor clinical outcomes in HCC, underscoring their potential utility as prognostic biomarkers and therapeutic targets.

## 4. Discussion

In this study, we systematically characterized the expression, prognostic relevance, regulatory networks, and biological associations of ZBTB40 in HCC. Our results demonstrated that ZBTB40 is significantly dysregulated in HCC and is associated with OS. Patients with high ZBTB40 expression exhibited distinct clinical outcomes, indicating that ZBTB40 expression is associated with prognostic outcomes in HCC. Furthermore, ZBTB40 expression is closely linked to multiple immune-related, epigenetic, and non-coding RNA regulatory mechanisms, suggesting that ZBTB40 expression is associated with multiple molecular features linked to HCC biology.

The present multi-omics investigation identified ZBTB40 as a gene associated with multiple molecular and clinical features in HCC, including epigenetic remodeling, transcriptional alterations, immune microenvironment characteristics, and ceRNA network associations [18,28]. Notably, ZBTB40 is overexpressed in HCC compared with normal liver tissue, displays consistent associations with poor OS and RFS, and exhibits multi-layered associations with promoter DNA hypomethylation, CNVs, immune infiltration profiles, and candidate ceRNA axes involving miR-24-3p, miR-132-3p, and miR-222-3p [52]. The convergence of these multi-omics observations suggests that ZBTB40 expression may reflect broader molecular alterations occurring in HCC rather than representing an isolated transcriptomic event [24].

Although ZBTB40 itself remains poorly characterized in oncology, its association with HCC-related molecular features may be biologically plausible given the known functions of ZBTB family proteins [52]. ZBTB transcription factors, such as BCL6 and PLZF, are canonical master regulators of cellular differentiation, proliferation, and apoptosis [29,53]. Mechanistically, these proteins utilize the C-terminal C2H2 zinc finger motifs for sequence-specific DNA binding, whereas their conserved N-terminal BTB/POZ domains facilitate the formation of homopolymeric or heteropolymeric structures [54]. ZBTB factors recruit coactivators or robust corepressor complexes via these PPIs, including N-CoR, SMRT, and histone deacetylases (HDACs), to specific chromatin loci, thereby orchestrating extensive chromatin remodeling and transcriptional regulation [55,56].

Although ZBTB40 itself has remained largely uncharacterized in oncology, our finding that it is overexpressed in HCC may provide a possible biological context for the observed associations identified in this study [6]. The observed promoter hypomethylation of ZBTB40 in tumor tissues, coupled with stage-associated CpG methylation dynamics, is potentially associated with altered transcriptional activity. The observed promoter hypomethylation of ZBTB40 in tumor tissues, coupled with stage-associated CpG methylation dynamics, may reflect altered transcriptional regulation. Similar epigenetic patterns have been broadly documented in HCC epigenomic landscapes [57].

The epigenetic and genomic remodeling associated with ZBTB40 further underscores its integration into cancer-relevant regulatory circuits [58,59]. Hypomethylation of promoter CpGs identified in HCC tumor samples is consistent with global DNA methylation alterations described in HCC, where widespread DNA hypomethylation can foster chromosomal instability and aberrant activation of oncogenic programs [60]. Additionally, stage-dependent shifts in CpG methylation, consistent with advanced tumor progression, suggest that epigenetic instability may be associated with transcriptional alterations observed during tumor progression [61]. Furthermore, the observed relationship between promoter demethylation and elevated ZBTB40 expression provides a potential explanatory framework for its increased expression in HCC [62].

The interaction between ZBTB40 and the immune microenvironment revealed a biologically relevant dimension of HCC pathobiology [63]. Correlations between ZBTB40 expression and the infiltration of cytotoxic (e.g., CD8^+^ T cells) and immunosuppressive (e.g., Tregs and macrophages) populations suggest that upregulation of this transcriptional hub may be associated with a complex immune contexture characterized by the coexistence of effector activation and immune suppression [4,27]. This duality is consistent with the dysfunctional immune microenvironment observed in HCC, where chronic inflammation and tumor-associated macrophage populations contribute to immune escape, whereas cytotoxic T cell infiltration may be stimulated by checkpoint pathways [64,65]. Therefore, these findings suggest an association between ZBTB40 expression and immune-related transcriptional signatures, although further mechanistic studies are required to clarify the biological basis of these observations [64,65,66].

The ceRNA network analysis further identified several non-coding RNA species potentially associated with ZBTB40-related expression patterns. miRNAs such as miR-24-3p, miR-132-3p, and miR-222-3p have previously been implicated in pathways related to proliferation, apoptosis, metastasis, and immune regulation in HCC [67]. Dysregulated miRNAs are recognized components of HCC pathogenesis and frequently participate in lncRNA–miRNA–mRNA interaction networks [67,68]. The concurrent identification of these axes in the present analysis suggests that ZBTB40 expression may be linked to broader RNA-based regulatory networks in HCC [69,70]. However, these interactions were computationally predicted and should be interpreted cautiously until experimentally validated.

From a clinical perspective, our findings suggest that ZBTB40 and its associated multi-omics features may have prognostic relevance in HCC. The consistent association between elevated ZBTB40 expression and unfavorable survival outcomes across multiple clinicopathological subgroups suggests that ZBTB40 expression may complement existing risk assessment approaches. Furthermore, the integration of expression, methylation, immune, and non-coding RNA features may provide a broader molecular context than single-biomarker approaches. Although directly targeting transcription factors remains challenging, the molecular associations identified in this study may inform future investigations of epigenetic or RNA-based therapeutic strategies. Epigenetic therapies targeting DNA methylation and chromatin accessibility, as well as RNA-based therapeutic approaches, are currently under investigation in HCC and other malignancies. Nevertheless, such applications remain speculative and require substantial experimental validation. In addition, the chemical–gene interaction signatures identified here require orthogonal validation before clinical interpretation.

Nevertheless, several limitations should be acknowledged. First, the analyses were retrospective and were derived predominantly from TCGA-based datasets, limiting direct clinical applicability without independent validation. Second, the biological roles of ZBTB40 and its associations with epigenetic, immune, and RNA regulatory features were inferred from bioinformatic analyses rather than experimentally validated functional studies. Third, although the identified ceRNA networks provide potentially informative hypotheses, these interactions remain computational predictions and require validation in cellular and animal models [69,70]. In addition, survival analyses were primarily based on TCGA-derived datasets and should therefore be considered hypothesis-generating until validated in independent cohorts. Future studies incorporating external cohorts and mechanistic validation will be necessary to clarify the biological and clinical significance of ZBTB40 in HCC.

## 5. Conclusions

This multi-omics analysis demonstrated that ZBTB40 expression is associated with multiple molecular features of HCC, including DNA methylation patterns, immune-related signatures, and predicted non-coding RNA networks. Elevated ZBTB40 expression was consistently associated with unfavorable clinical outcomes, supporting its potential relevance as a prognostic biomarker candidate. These findings provide a comprehensive bioinformatic framework for future experimental studies investigating the biological and clinical significance of ZBTB40 in HCC.

## Figures and Tables

**Table 1:** Survival analysis of ZBTB40 expression in HCC.

Clinicopathological Characteristics	Overall Survival			Relapse-Free Survival			Progression-Free Survival			Disease-Specific Survival		
	(*n* = 364)			(*n* = 316)			(*n* = 370)			(*n* = 362)		
	*N*	HR	*p*-Value	*N*	HR	*p*-Value	*N*	HR	*p*-Value	*N*	HR	*p*-Value
**SEX**												
Male	246	2.52	**0.000056**	210	2.02	**0.00053**	249	1.77	**0.0017**	244	2.61	**0.001**
		(1.58–4.01)			(1.35–3.02)			(1.24–2.55)			(1.44–4.71)	
Female	118	1.1	0.73	106	1.5	0.18	121	1.82	**0.021**	118	1.17	0.66
		(0.63–1.92)			(0.83–2.7)			(1.09–3.07)			(0.58–2.39)	
**STAGE**												
I	170	1.78	0.065	153	1.17	0.57	171	1.5	0.11	168	1.96	0.13
		(0.96–3.3)			(0.68–2)			(0.91–2.47)			(0.8–4.78)	
I + II	253	2.08	**0.003**	228	1.67	**0.016**	256	1.97	**0.00042**	251	3.11	**0.0017**
		(1.27–3.42)			(1.1–2.54)			(1.34–2.9)			(1.47–6.54)	
II	83	2.81	0.012	75	1.43	0.29	85	1.83	**0.048**	83	7.03	**0.0032**
		(1.22–6.49)			(0.74–2.79)			(1–3.36)			(1.55–31.78)	
II + III	166	2.06	**0.0026**	145	1.52	0.063	170	1.57	**0.025**	166	2.33	**0.0054**
		(1.28–3.32)			(0.97–2.38)			(1.06–2.34)			(1.26–4.29)	
III	83	1.53	0.16	70	1.55	0.15	85	1.2	0.5	83	1.69	0.15
		(0.84–2.78)			(0.85–2.82)			(0.7–2.06)			(0.82–3.49)	
III + IV	87	1.54	0.14	70	1.55	0.15	90	1.2	0.5	87	1.79	0.1
		(0.86–2.74)			(0.85–2.82)			(0.71–2.02)			(0.89–3.61)	
IV	4	-	-	0	-	-	5	-	-	3	-	-
**GRADE**												
I	55	2.29	0.082	45	0.8	0.66	55	1.39	0.41	55	1.93	0.27
		(0.88–5.96)			(0.3–2.13)			(0.63–3.04)			(0.58–6.42)	
II	174	1.54	0.097	149	2.12	**0.003**	177	1.91	**0.0037**	171	1.94	**0.049**
		(0.92–2.59)			(1.28–3.53)			(1.22–2.97)			(0.99–3.82)	
III	118	2.45	**0.0039**	107	1.92	**0.016**	121	2.11	**0.0031**	119	2. 8	**0.0076**
		(1.31–4.59)			(1.12–3.3)			(1.27–3.5)			(1.27–6.17)	
IV	12	-	-	11	-	-	12	-	-	12	-	-
**AJCC_T**												
I	180	1.81	**0.045**	160	1.2	0.5	181	1.53	0.084	178	1.97	0.097
		(1.01–3.27)			(0.71–2.03)			(0.94–2.47)			(0.87–4.44)	
II	90	2.42	**0.019**	80	1.46	0.24	93	1.8	**0.035**	91	3.93	0.01
		(1.13–5.19)			(0.77–2.76)			(1.03–3.14)			(1.28–12.06)	
III	78	1.64	0.11	67	1.49	0.2	80	1.27	0.41	77	1.61	0.21
		(0.88–3.02)			(0.8–2.79)			(0.72–2.22)			(0.76–3.4)	
IV	13	-	-	6	-	-	13	-	-	13	-	-
**Vascular invasion**												
None	203	1.64	0.061	175	1.52	0.09	205	1.68	**0.022**	201	1.65	0.17
		(0.97–2.75)			(0.93–2.46)			(1.07–2.64)			(0.8–3.37)	
Micro	90	1.35	0.44	82	1.34	0.36	92	1.72	0.059	90	1.12	0.84
		(0.63–2.88)			(0.71–2.53)			(0.97–3.06)			(0.37–3.34)	
Macro	16	-	-	14	-	-	16	-	-	14	-	-
**RACE**												
White	181	1.19	0.46	147	1.33	0.22	184	1.47	0.057	179	1.48	0.17
		(0.75–1.88)			(0.84–2.09)			(0.99–2.18)			(0.85–2.61)	
Asian	155	2.92	**0.00064**	145	1.83	**0.018**	157	1.98	**0.0044**	154	3.16	**0.005**
		(1.53–5.56)			(1.1–3.06)			(1.23–3.2)			(1.36–7.37)	
**Alcohol consumption**												
Yes	115	1.59	0.15	99	2.57	**0.0014**	117	2.15	**0.0034**	117	2.11	**0.041**
		(0.84–2.98)			(1.41–4.69)			(1.27–3.64)			(1.02–4.38)	
None	202	1.76	**0.016**	183	1.48	0.08	205	1.69	**0.01**	199	2.33	**0.0076**
		(1.11–2.8)			(0.95–2.31)			(1.13–2.53)			(1.23-4.42)	
**Hepatitis virus**												
Yes	150	2.73	**0.0032**	139	1.67	**0.041**	153	1.94	**0.0048**	151	3.17	**0.0077**
		(1.36–5.48)			(1.02–2.75)			(1.21–3.1)			(1.3–7.75)	
None	167	1.24	0.35	143	1.81	**0.02**	169	1.8	**0.008**	165	1.65	0.082
		(0.79–1.94)			(1.09–3)			(1.16–2.8)			(0.93–2.91)	

**Table 2:** Chemicals associated with altered ZBTB40 expression.

Chemical Name	Chemical ID	Interaction Actions
4-(4-((5-(4,5-dimethyl-2-nitrophenyl)-2-furanyl)methylene)-4,	C584509	increases expression
5-dihydro-3-methyl-5-oxo-1H-pyrazol-1-yl)benzoic acid		
4-hydroxyphenyl 4-isopropoxyphenylsulfone	C000613560	increases expression
abrine	C496492	increases expression
Acrylamide	D020106	increases expression
Aflatoxin B1	D016604	increases expression
aristolochic acid I	C000228	increases expression
Beclomethasone	D001507	increases expression
Cadmium Chloride	D019256	increases expression
Carbon Tetrachloride	D002251	increases expression
Copper Sulfate	D019327	increases expression
deoxynivalenol	C007262	increases expression
dicrotophos	C000944	increases expression
Diethylhexyl Phthalate	D004051	increases expression
Gentamicins	D005839	increases expression
ICG 001	C492448	increases expression
Methyl Methanesulfonate	D008741	increases expression
Silicon Dioxide	D012822	increases expression
sodium arsenite	C017947	increases expression
Sorafenib	D000077157	increases expression
T-2 Toxin	D013605	increases expression
Zeranol	D015029	increases expression
Amphetamine	D000661	decreases expression
Atrazine	D001280	decreases expression
bisphenol A	C006780	decreases expression
bisphenol S	C543008	decreases expression
decamethrin	C017180	decreases expression
Dietary Fats	D004041	decreases expression
Doxorubicin	D004317	decreases expression
epoxiconazole	C109476	decreases expression
Folic Acid	D005492	decreases expression
Methamphetamine	D008694	decreases expression
N-(2-(1,1′-bicyclopropyl)-2-ylphenyl)-3-(difluoromethyl)-1-methyl-1H-pyrazole-4-carboxamide	C583365	decreases expression
Nanotubes, Carbon	D037742	decreases expression
Particulate Matter	D052638	decreases expression
pirinixic acid	C006253	decreases expression
Tetrachlorodibenzodioxin	D013749	decreases expression
Thioacetamide	D013853	decreases expression
Tretinoin	D014212	decreases expression
Vorinostat	D000077337	decreases expression

**Table 3:** Chemical–gene interaction similarity analysis of ZBTB40.

Gene	Similarity Index	Common Interacting Chemicals
SAP30BP	0.38393	43
TNK2	0.38333	46
ADAMTSL5	0.38182	42
PPP1R16A	0.38	38
SMYD4	0.37963	41
CAMSAP1	0.37719	43
TPRKB	0.37615	41
RAB43	0.37607	44
ZHX3	0.375	45
RNF13	0.36923	48
CCNY	0.36752	43
SFXN5	0.36667	44
KCTD20	0.36634	37
UBR3	0.36207	42
NT5DC3	0.35965	41
KDM2A	0.35897	42
CHD3	0.35821	48
IFT122	0.35714	40
PARP6	0.35644	36
NOP14	0.35455	39
TMEM214	0.35455	39

## Data Availability

All data are available upon reasonable request from the corresponding author.

## Supplementary Materials

The following supporting information can be downloaded at: https://www.mdpi.com/article/10.3390/medicina62071244/s1. Figure S1. ZBTB40-associated with hsa-miR-24-3p network in LIHC. Interaction networks of hsa-miR-24-3p with lncRNAs (A), circRNAs (B), pseudogenes (C), and sncRNAs (D). (E) Expression of hsa-miR-24-3p-associated lncRNAs in LIHC and normal liver tissues. (F) Overall survival of hsa-miR-24-3p-associated with lncRNAs. (G) Expression of hsa-miR-24-3p-associated circRNAs in LIHC. (H) Overall survival for hsa-miR-24-3p-associated circRNAs. Figure S2. ZBTB40-associated with hsa-miR-34a-5p network in LIHC. Interaction networks of hsa-miR-34a-5p with lncRNAs (A), circRNAs (B), pseudogenes (C), and sncRNAs (D). (E) Expression of hsa-miR-34a-5p-associated lncRNAs in LIHC and normal liver tissues. (F) Overall survival of hsa-miR-34a-5p-associated with lncRNAs. (G) Expression of hsa-miR-34a-5p-associated circRNAs in LIHC. (H) Overall survival for hsa-miR-34a-5p-associated circRNAs. Figure S3. ZBTB40-associated with hsa-miR-132-3p network in LIHC. Interaction networks of hsa-miR-132-3p with lncRNAs (A), circRNAs (B), pseudogenes (C), and sncRNAs (D). (E) Expression of hsa-miR-132-3p-associated lncRNAs in LIHC and normal liver tissues. (F) Overall survival of hsa-miR-132-3p-associated with lncRNAs. (G) Expression for hsa-miR-132-3p-associated circRNAs in LIHC. (H) Overall survival of hsa-miR-132-3p-associated circRNAs. Figure S4. ZBTB40-associated with hsa-miR-222-3p network in LIHC. Interaction networks of hsa-miR-222-3p with lncRNAs (A), circRNAs (B), pseudogenes (C), and sncRNAs (D). (E) Expression of hsa-miR-222-3p-associated lncRNAs in LIHC and normal liver tissues. (F) Overall survival of hsa-miR-222-3p-associated with lncRNAs. (G) Expression for hsa-miR-222-3p-associated circRNAs in LIHC. (H) Overall survival of hsa-miR-222-3p-associated circRNAs. Figure S5. External validation of ZBTB40 expression and prognostic relevance in the HCCDB6/GSE14520 cohort. Table S1. Figure 9 miRNA hsa-miR-24-3p; Table S2. Figure 10 hsa-miR-34a-5p; Table S3. Figure 11 hsa-miR-132-3p; Table S4. Figure 12 hsa-miR-222-3p; Table S5. Multivariable Cox regression analysis of ZBTB40 in LIHC using the TCGA-LIHC cohort (n = 371).

## Abbreviations

The following abbreviations are used in this manuscript:

ceRNA	Competing endogenous RNA
CI	Confidence interval
CNV	Copy number variation
CTD	Comparative Toxicogenomics Database
DFI	Disease-free interval
DSS	Disease-specific survival
FDR	False discovery rate
GO	Gene Ontology
GSCA	Gene Set Cancer Analysis
GSEA	Gene Set Enrichment Analysis
HDAC	Histone deacetylase
HR	Hazard ratio
KEGG	Kyoto Encyclopedia of Genes and Genomes
HCC	Hepatocellular Carcinoma
ncRNA	Non-coding RNA
OS	Overall survival
PFS	Progression-free survival
PPI	Protein–protein interaction
TCGA	The Cancer Genome Atlas
Th	T helper cell
Treg	Regulatory T cell
ZBTB40	Zinc finger and BTB domain-containing protein 40
