# Supplementary material for: Integrated Bioinformatics and Multi-Omics Analysis of ZBTB40 Expression, Prognostic Relevance, and Regulatory Networks in Hepatocellular Carcinoma
Source: Medicina (Kaunas). 2026 Jun 27;62(7):1244. doi: 10.3390/medicina62071244 (PMC13413636; doi:10.3390/medicina62071244)
Supplement: Supplementary file 1 [file medicina-62-01244-s001.zip › medicina-4302238-supplementary.pdf]

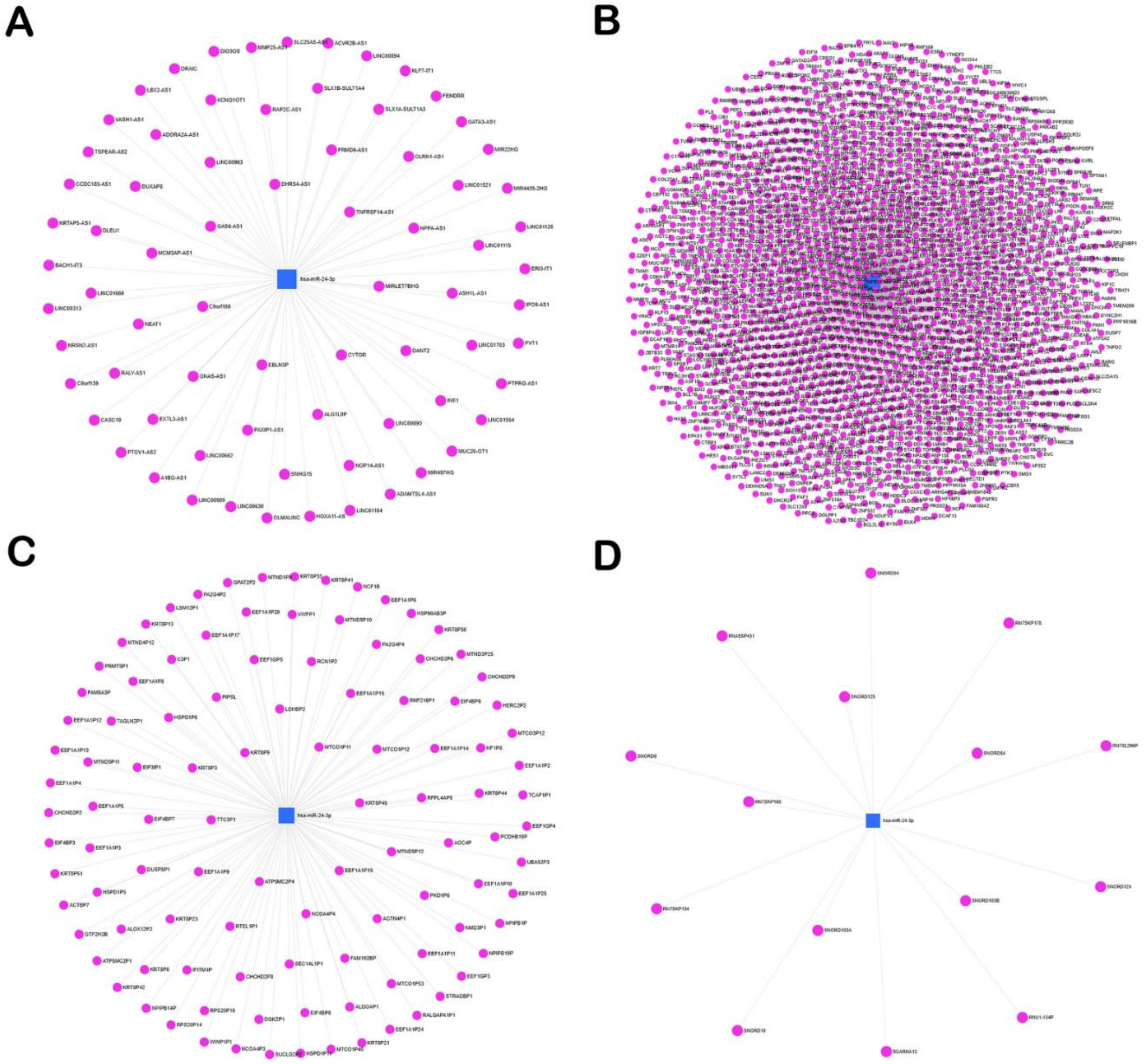
A
B
C
D
hsa-miR-24-3p

E

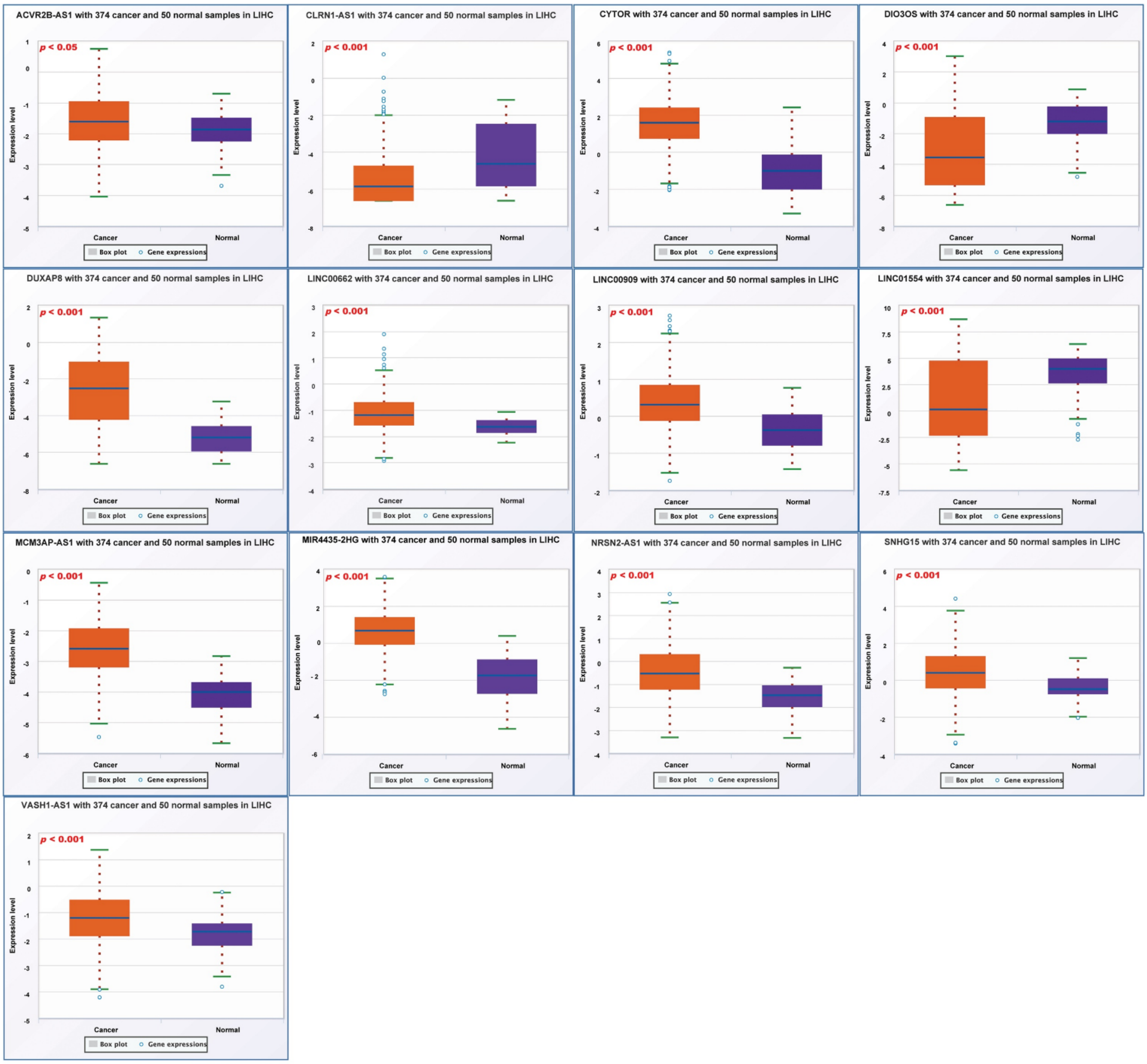
ACVR2B-AS1 with 374 cancer and 50 normal samples in LIHC
p < 0.05
CLRN1-AS1 with 374 cancer and 50 normal samples in LIHC
p < 0.001
CYTOR with 374 cancer and 50 normal samples in LIHC
p < 0.001
DIO3OS with 374 cancer and 50 normal samples in LIHC
p < 0.001
DUXAP8 with 374 cancer and 50 normal samples in LIHC
p < 0.001
LINC00662 with 374 cancer and 50 normal samples in LIHC
p < 0.001
LINC00909 with 374 cancer and 50 normal samples in LIHC
p < 0.001
LINC01554 with 374 cancer and 50 normal samples in LIHC
p < 0.001
MCM3AP-AS1 with 374 cancer and 50 normal samples in LIHC
p < 0.001
MIR4435-2HG with 374 cancer and 50 normal samples in LIHC
p < 0.001
NRSN2-AS1 with 374 cancer and 50 normal samples in LIHC
p < 0.001
SNHG15 with 374 cancer and 50 normal samples in LIHC
p < 0.001
VASH1-AS1 with 374 cancer and 50 normal samples in LIHC
p < 0.001
Expression level
Cancer
Normal
Box plot
Gene expressions

F

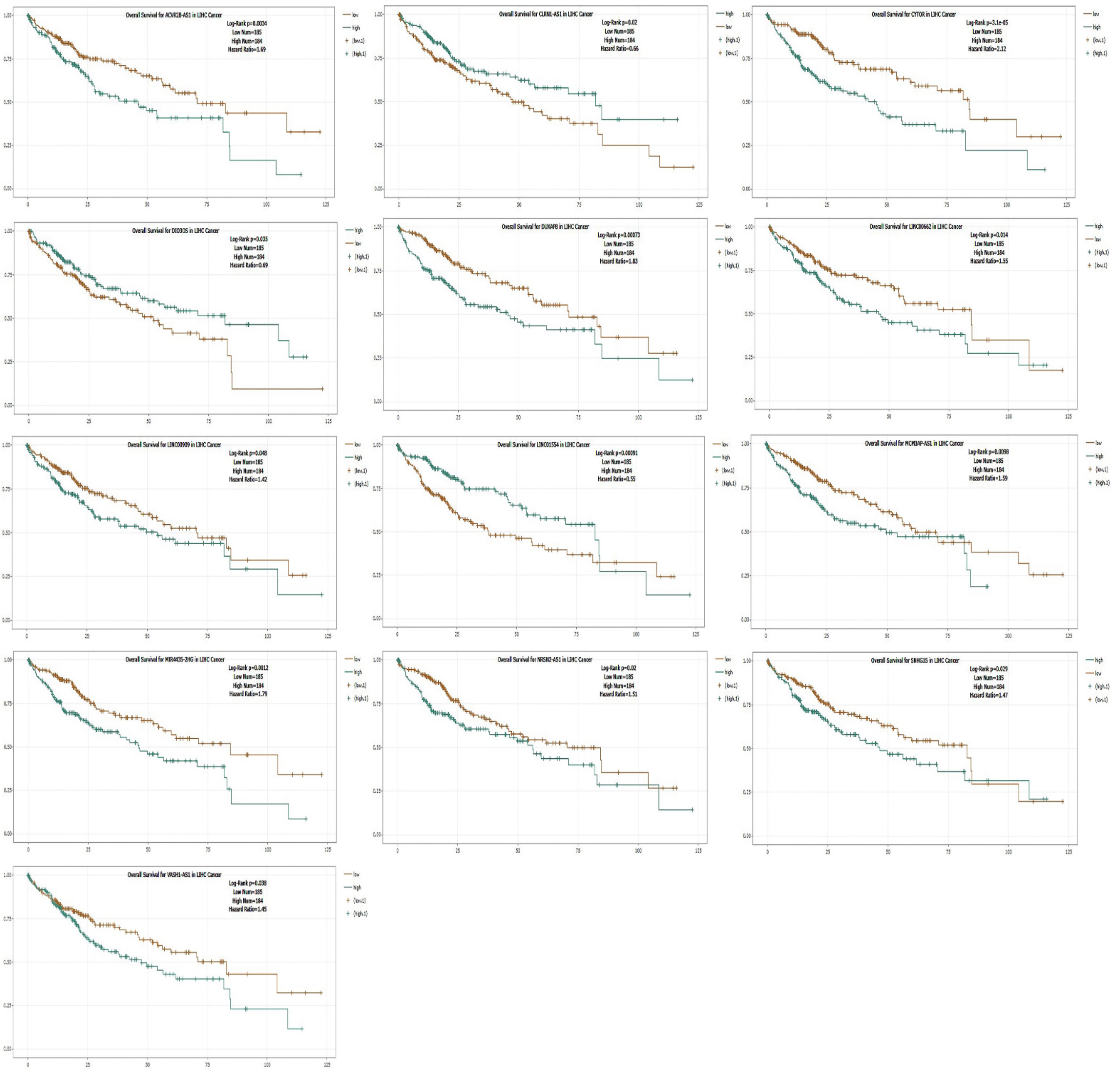
Overall Survival for ACVR2B-AS1 in LIHC Cancer
Log-Rank p=0.0034
Low Num=185
High Num=184
Hazard Ratio=1.69
Overall Survival for CLRN1-AS1 in LIHC Cancer
Log-Rank p=0.02
Low Num=185
High Num=184
Hazard Ratio=0.66
Overall Survival for CYTOR in LIHC Cancer
Log-Rank p=3.1e-05
Low Num=185
High Num=184
Hazard Ratio=2.12
Overall Survival for DIO3OS in LIHC Cancer
Log-Rank p=0.035
Low Num=185
High Num=184
Hazard Ratio=0.69
Overall Survival for DUXAP8 in LIHC Cancer
Log-Rank p=0.00073
Low Num=185
High Num=184
Hazard Ratio=1.83
Overall Survival for LINC00662 in LIHC Cancer
Log-Rank p=0.014
Low Num=185
High Num=184
Hazard Ratio=1.55
Overall Survival for LINC00909 in LIHC Cancer
Log-Rank p=0.048
Low Num=185
High Num=184
Hazard Ratio=1.42
Overall Survival for LINC01554 in LIHC Cancer
Log-Rank p=0.00091
Low Num=185
High Num=184
Hazard Ratio=0.55
Overall Survival for MCM3AP-AS1 in LIHC Cancer
Log-Rank p=0.0098
Low Num=185
High Num=184
Hazard Ratio=1.59
Overall Survival for MIR4435-2HG in LIHC Cancer
Log-Rank p=0.0012
Low Num=185
High Num=184
Hazard Ratio=1.79
Overall Survival for NRSN2-AS1 in LIHC Cancer
Log-Rank p=0.02
Low Num=185
High Num=184
Hazard Ratio=1.51
Overall Survival for SNHG15 in LIHC Cancer
Log-Rank p=0.029
Low Num=185
High Num=184
Hazard Ratio=1.47
Overall Survival for VASH1-AS1 in LIHC Cancer
Log-Rank p=0.038
Low Num=185
High Num=184
Hazard Ratio=1.45

G

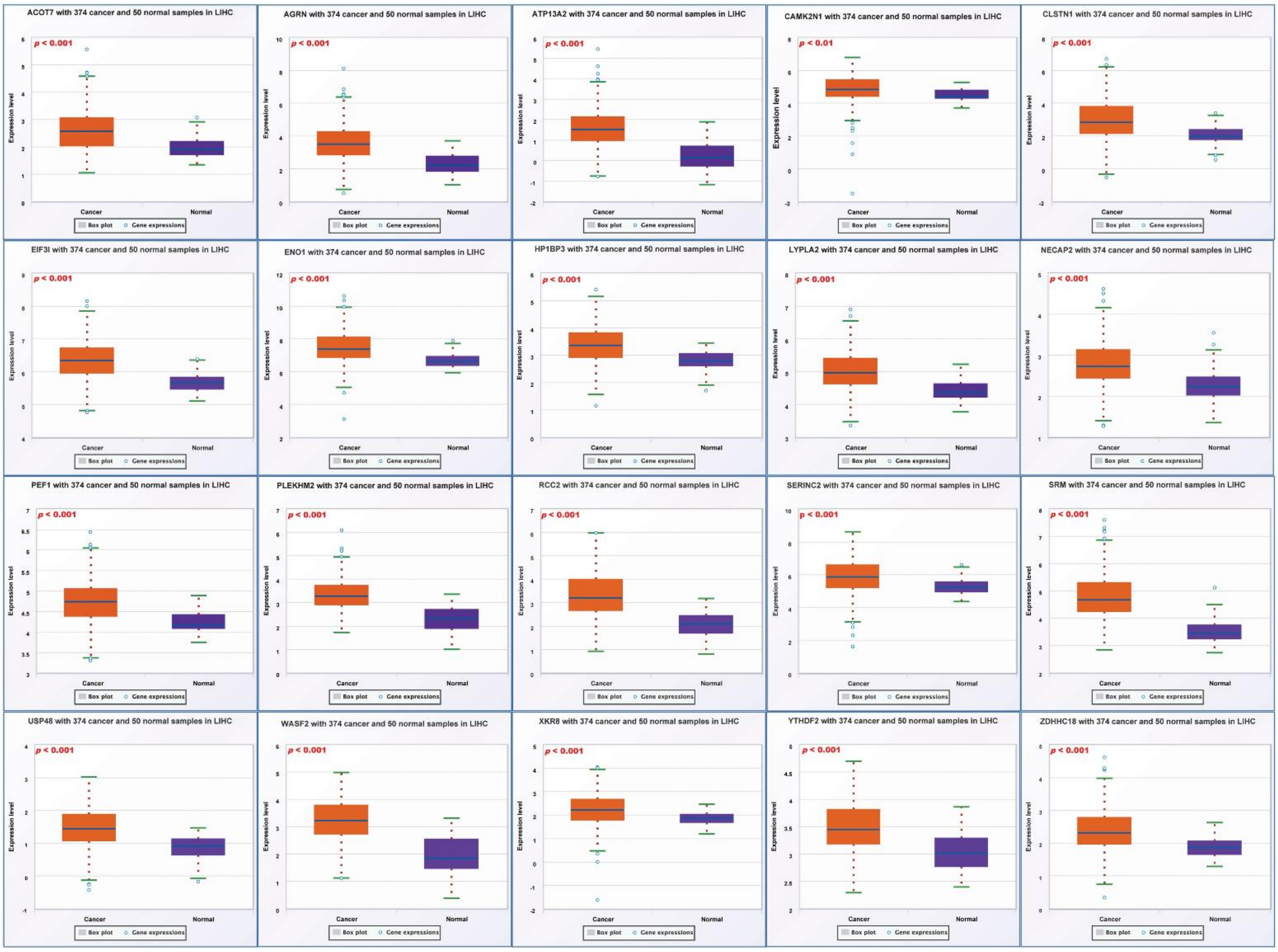
ACOT7 with 374 cancer and 50 normal samples in LIHC
p < 0.001
AGRN with 374 cancer and 50 normal samples in LIHC
p < 0.001
ATP13A2 with 374 cancer and 50 normal samples in LIHC
p < 0.001
CAMK2N1 with 374 cancer and 50 normal samples in LIHC
p < 0.01
CLSTN1 with 374 cancer and 50 normal samples in LIHC
p < 0.001
EIF3I with 374 cancer and 50 normal samples in LIHC
p < 0.001
ENO1 with 374 cancer and 50 normal samples in LIHC
p < 0.001
HP1BP3 with 374 cancer and 50 normal samples in LIHC
p < 0.001
LYPLA2 with 374 cancer and 50 normal samples in LIHC
p < 0.001
NECAP2 with 374 cancer and 50 normal samples in LIHC
p < 0.001
PEF1 with 374 cancer and 50 normal samples in LIHC
p < 0.001
PLEKHM2 with 374 cancer and 50 normal samples in LIHC
p < 0.001
RCC2 with 374 cancer and 50 normal samples in LIHC
p < 0.001
SERINC2 with 374 cancer and 50 normal samples in LIHC
p < 0.001
SRM with 374 cancer and 50 normal samples in LIHC
p < 0.001
USP48 with 374 cancer and 50 normal samples in LIHC
p < 0.001
WASF2 with 374 cancer and 50 normal samples in LIHC
p < 0.001
XKR8 with 374 cancer and 50 normal samples in LIHC
p < 0.001
YTHDF2 with 374 cancer and 50 normal samples in LIHC
p < 0.001
ZDHHC18 with 374 cancer and 50 normal samples in LIHC
p < 0.001
Expression level
Cancer
Normal
Box plot
Gene expressions

H

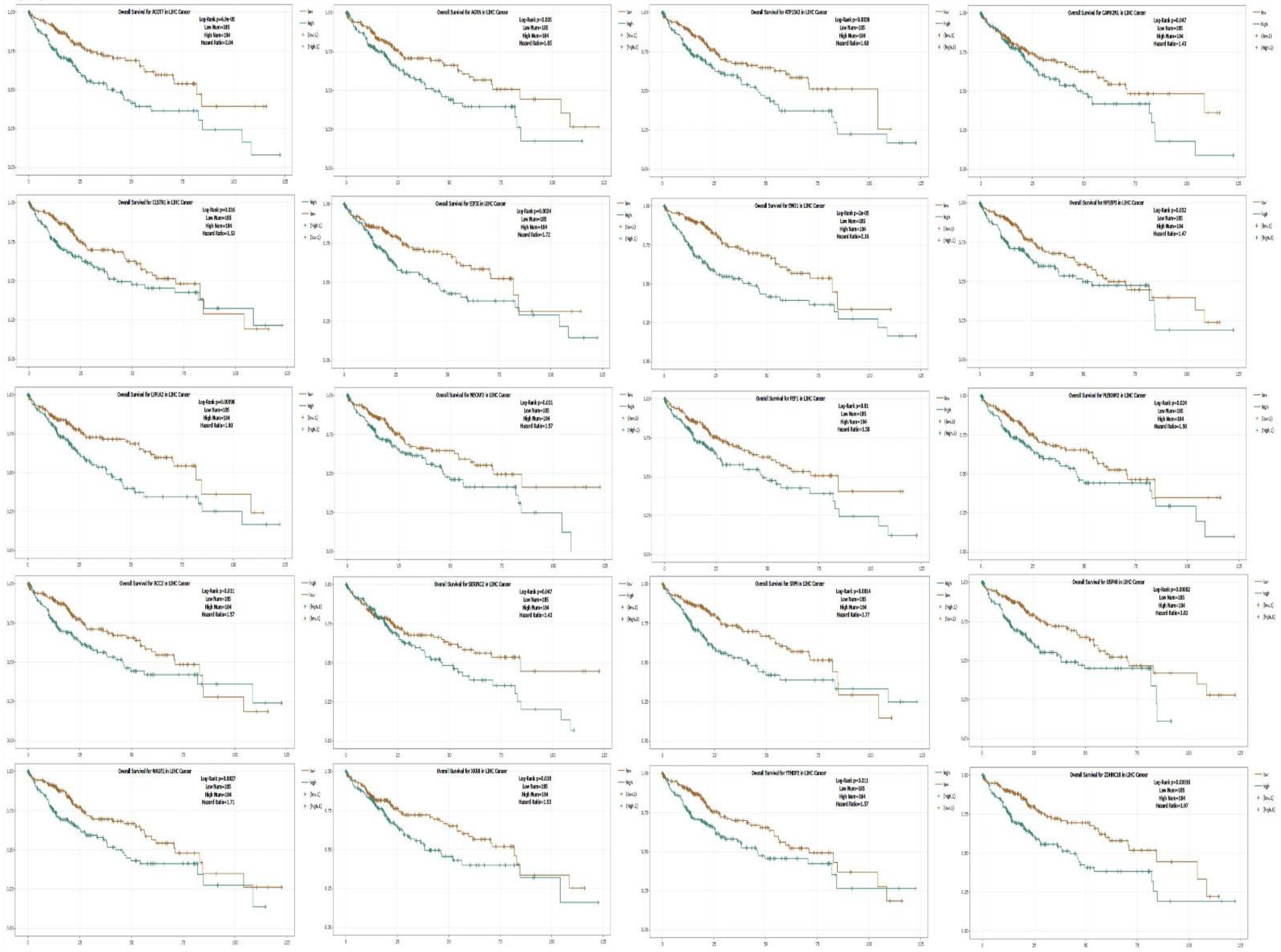

Supplementary Figure S1. ZBTB40-associated with hsa-miR-24-3p network in LIHC. Interaction networks of hsa-miR-24-3p with lncRNAs (A), circRNAs (B), pseudogenes (C), and sncRNAs (D). (E) Expression of hsa-miR-24-3p-associated lncRNAs in LIHC and normal liver tissues. (F) Overall survival of hsa-miR-24-3p-associated with lncRNAs. (G) Expression of hsa-miR-24-3p-associated circRNAs in LIHC. (H) Overall survival for hsa-miR-24-3p-associated circRNAs.

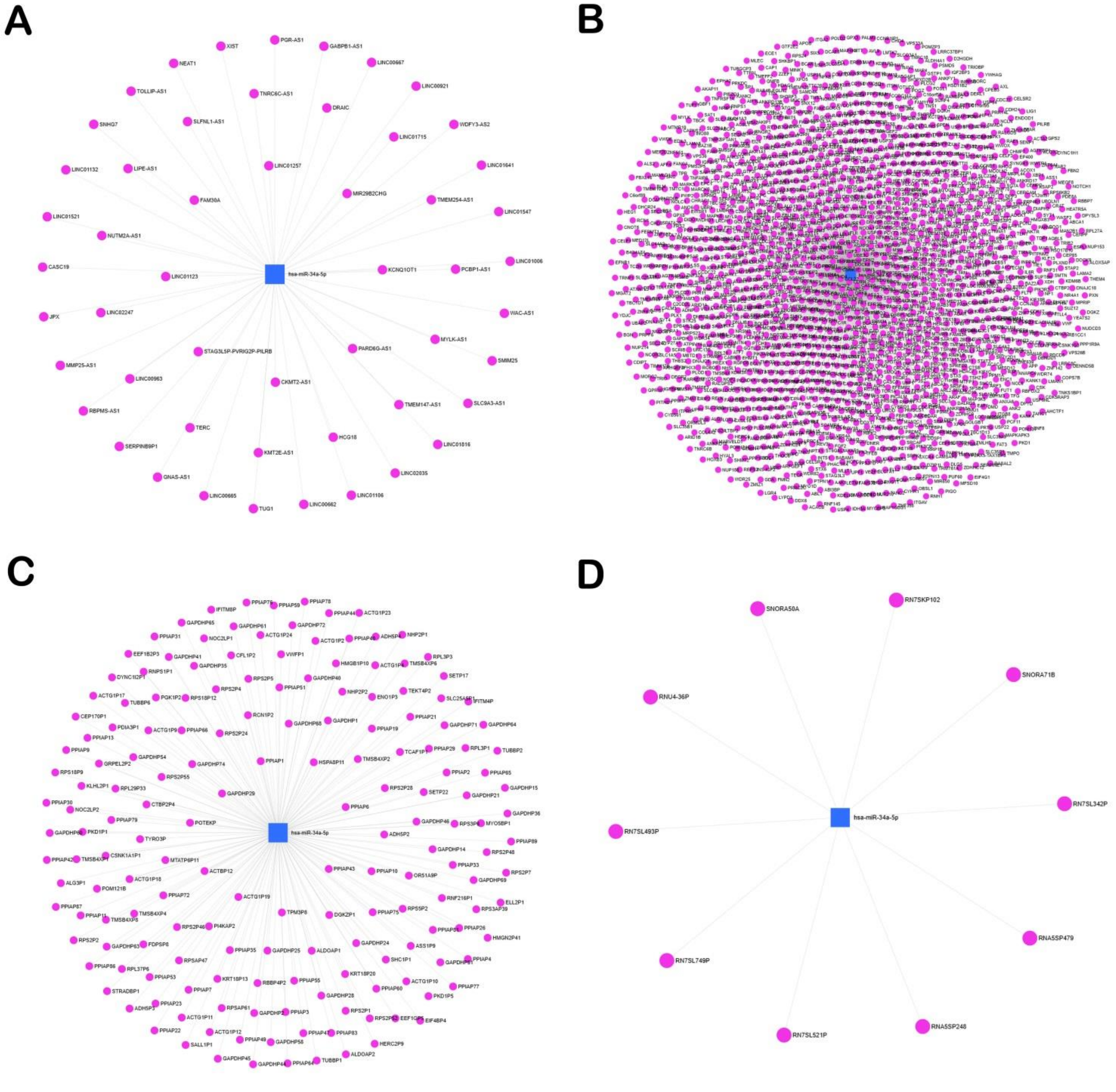
A
B
C
D
hsa-miR-34a-5p
XIST
PQR-AS1
GABPB1-AS1
NEAT1
LINC00667
TOLLIP-AS1
TNRC6C-AS1
LINC00921
DRAIC
SNHG7
SLFNL1-AS1
WDFY3-AS2
LINC01715
LINC01132
LIPE-AS1
LINC01257
LINC01641
MIR29B2CHG
FAM30A
TMEM254-AS1
LINC01521
LINC01547
NUTM2A-AS1
CASC19
LINC01123
KCNQ1OT1
PCBP1-AS1
LINC01006
JPX
LINC02247
WAC-AS1
MYLK-AS1
STAG3L5P-PVRIG2P-PILRB
PARD6G-AS1
MMP25-AS1
SMIM25
LINC00963
CKMT2-AS1
RBPMS-AS1
TMEM147-AS1
SLC9A3-AS1
TERC
HCG18
SERPINB9P1
LINC01016
KMT2E-AS1
GNAS-AS1
LINC02035
LINC00665
LINC01106
TUG1
LINC00662
SNORA50A
RN7SKP102
SNORA71B
RNU4-36P
RN7SL342P
RN7SL493P
RNA5SP479
RN7SL749P
RNA5SP248
RN7SL521P

E

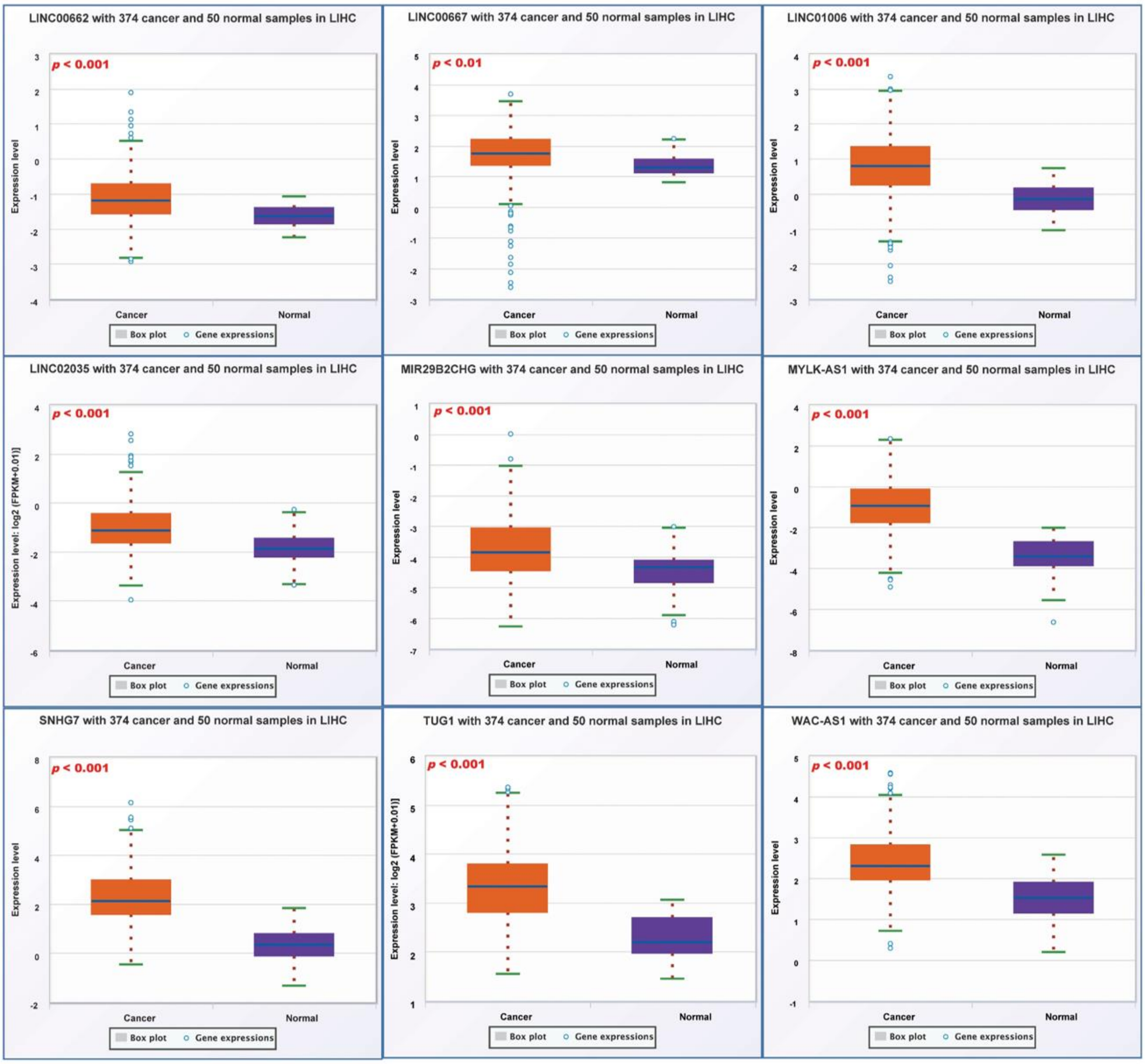
LINC00662 with 374 cancer and 50 normal samples in LIHC
p < 0.001
LINC00667 with 374 cancer and 50 normal samples in LIHC
p < 0.01
LINC01006 with 374 cancer and 50 normal samples in LIHC
p < 0.001
LINC02035 with 374 cancer and 50 normal samples in LIHC
p < 0.001
MIR29B2CHG with 374 cancer and 50 normal samples in LIHC
p < 0.001
MYLK-AS1 with 374 cancer and 50 normal samples in LIHC
p < 0.001
SNHG7 with 374 cancer and 50 normal samples in LIHC
p < 0.001
TUG1 with 374 cancer and 50 normal samples in LIHC
p < 0.001
WAC-AS1 with 374 cancer and 50 normal samples in LIHC
p < 0.001
Expression level
Expression level: log2 (FPKM+0.01)]
Cancer
Normal
Box plot
Gene expressions

F

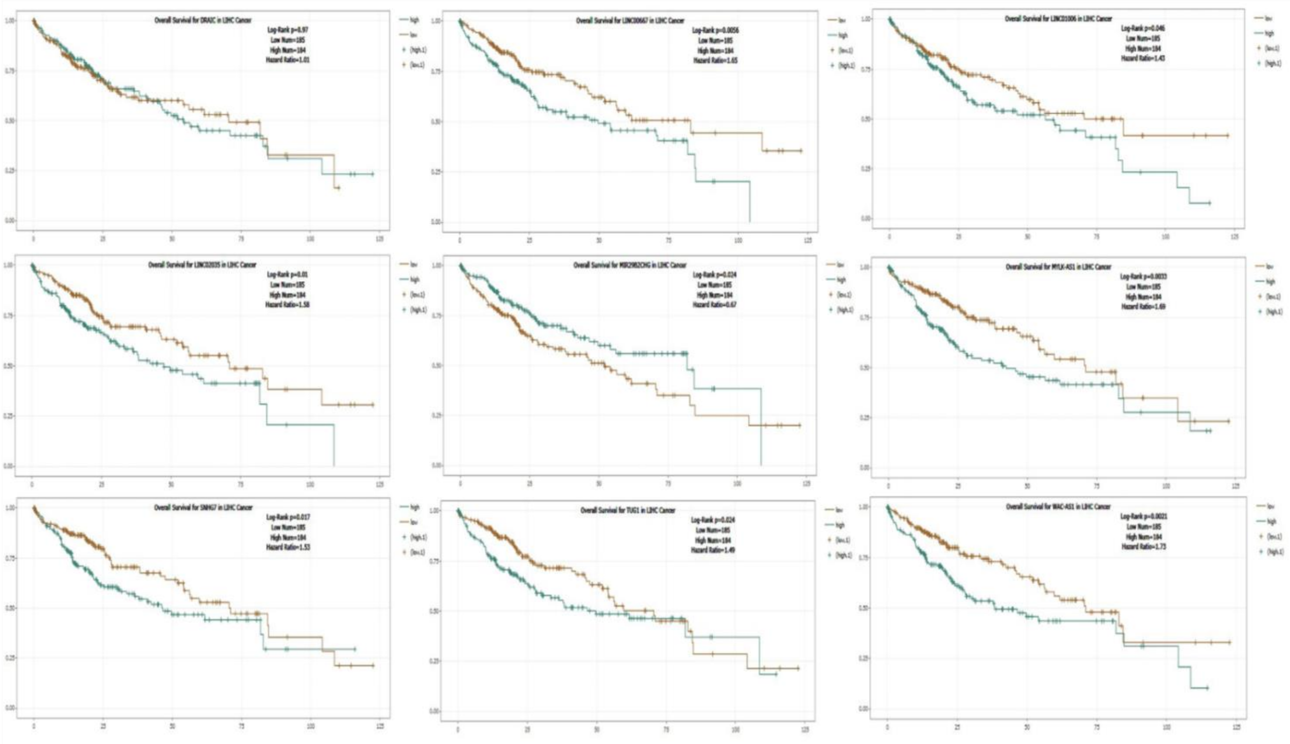

G

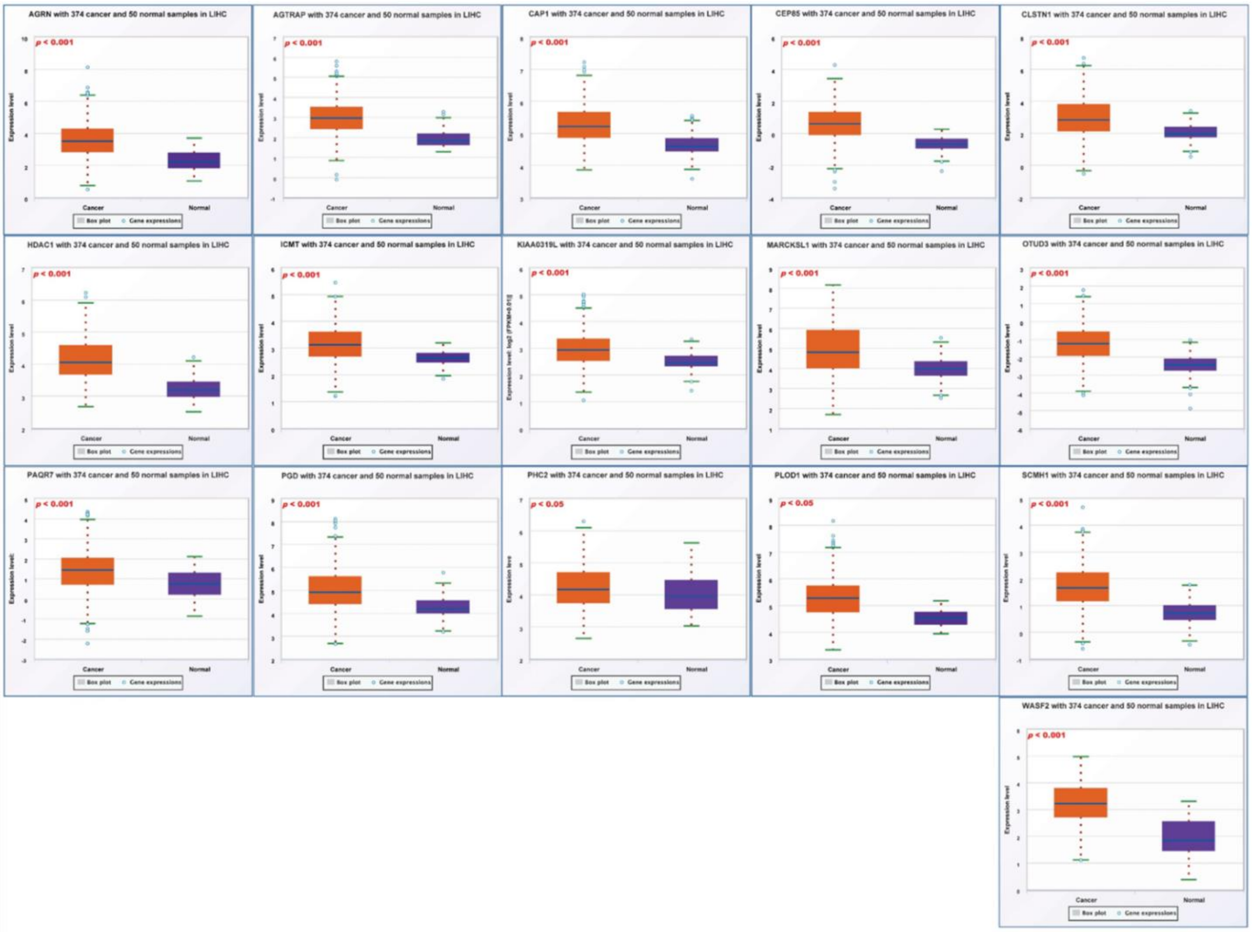
AGRN with 374 cancer and 50 normal samples in LIHC
AGTRAP with 374 cancer and 50 normal samples in LIHC
CAP1 with 374 cancer and 50 normal samples in LIHC
CEP85 with 374 cancer and 50 normal samples in LIHC
CLSTN1 with 374 cancer and 50 normal samples in LIHC
HDAC1 with 374 cancer and 50 normal samples in LIHC
ICMT with 374 cancer and 50 normal samples in LIHC
KIAA0319L with 374 cancer and 50 normal samples in LIHC
MARCKSL1 with 374 cancer and 50 normal samples in LIHC
OTUD3 with 374 cancer and 50 normal samples in LIHC
PAQR7 with 374 cancer and 50 normal samples in LIHC
PGD with 374 cancer and 50 normal samples in LIHC
PHC2 with 374 cancer and 50 normal samples in LIHC
PLOD1 with 374 cancer and 50 normal samples in LIHC
SCMH1 with 374 cancer and 50 normal samples in LIHC
WASF2 with 374 cancer and 50 normal samples in LIHC
p < 0.001
p < 0.05
Expression level
Cancer
Normal
Box plot
Gene expressions

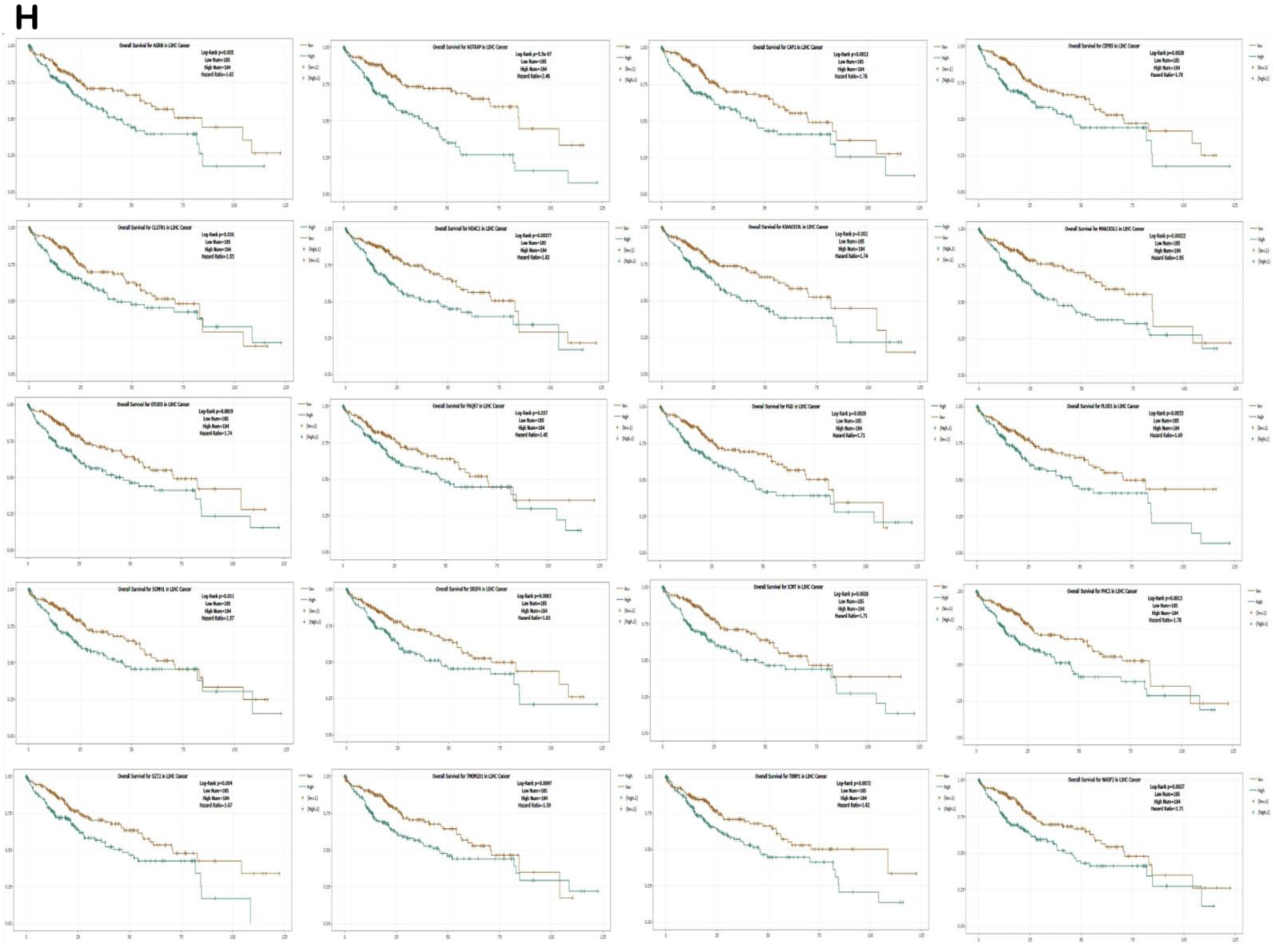

Supplementary Figure S2. ZBTB40-associated with hsa-miR-34a-5p network in LIHC. Interaction networks of hsa-miR-34a-5p with lncRNAs (A), circRNAs (B), pseudogenes (C), and sncRNAs (D). (E) Expression of hsa-miR-34a-5p-associated lncRNAs in LIHC and normal liver tissues. (F) Overall survival of hsa-miR-34a-5p-associated with lncRNAs. (G) Expression of hsa-miR-34a-5p-associated circRNAs in LIHC. (H) Overall survival for hsa-miR-34a-5p-associated circRNAs.

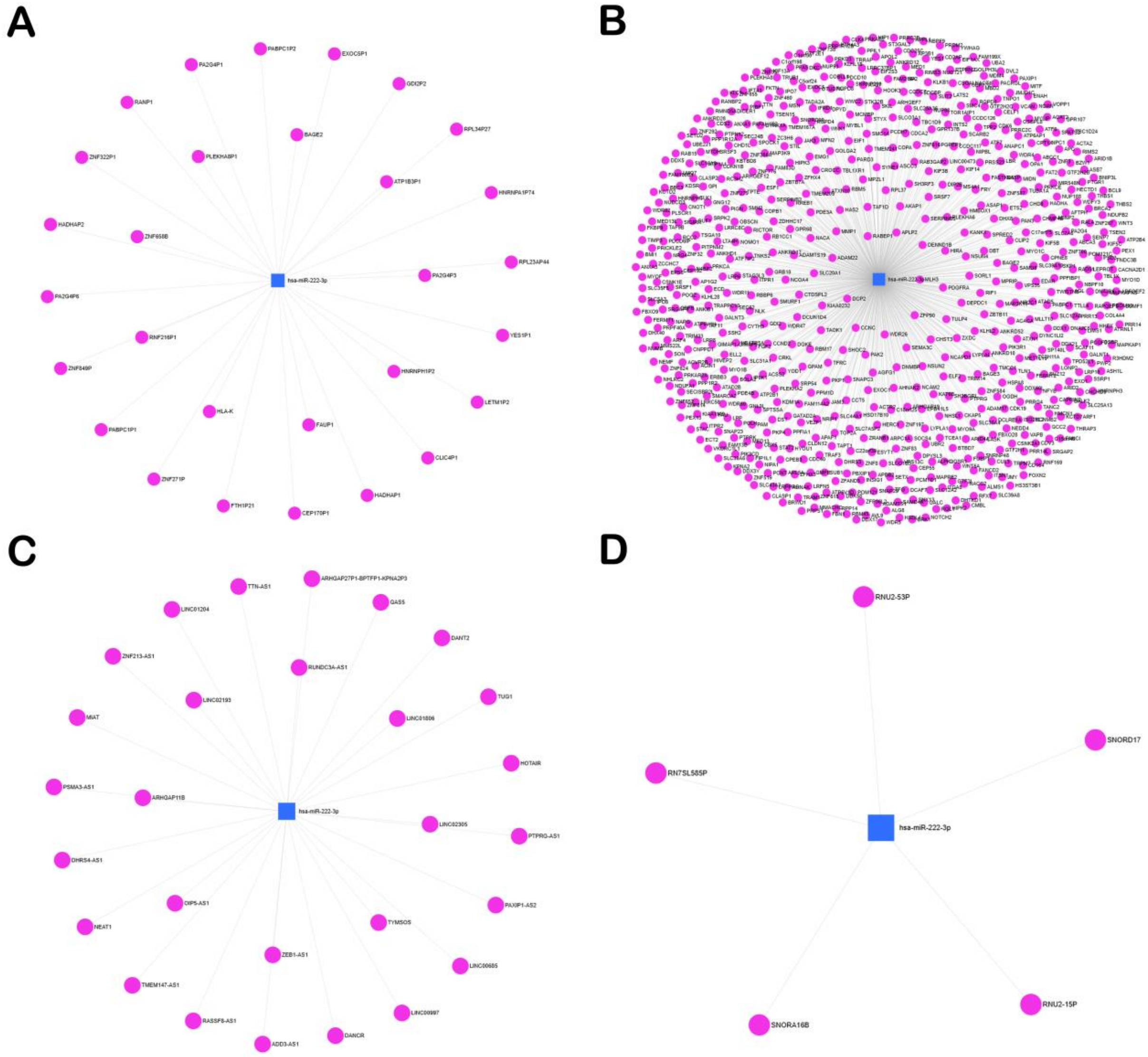

A
PABPC1P2
EXOC5P1
PA2G4P1
GDI2P2
RANP1
BAGE2
RPL34P27
ZNF322P1
PLEKHA8P1
ATP1B3P1
HNRNPA1P74
HADHAP2
ZNF658B
RPL23AP44
hsa-miR-222-3p
PA2G4P3
PA2G4P6
YES1P1
RNF216P1
ZNF849P
HNRNPH1P2
LETM1P2
HLA-K
FAUP1
PABPC1P1
CLIC4P1
ZNF271P
HADHAP1
FTH1P21
CEP170P1
B
C
ARHGAP27P1-BPTFP1-KPNA2P3
TTN-AS1
GAS5
LINC01204
DANT2
ZNF213-AS1
RUNDC3A-AS1
LINC02193
TUG1
MIAT
LINC01806
HOTAIR
PSMA3-AS1
ARHGAP11B
hsa-miR-222-3p
LINC02305
PTPRG-AS1
DHRS4-AS1
OIP5-AS1
PAXIP1-AS2
NEAT1
TYMSOS
ZEB1-AS1
LINC00685
TMEM147-AS1
LINC00997
RASSF8-AS1
DANCR
ADD3-AS1
D
RNU2-53P
SNORD17
RN7SL585P
hsa-miR-222-3p
RNU2-15P
SNORA16B

E

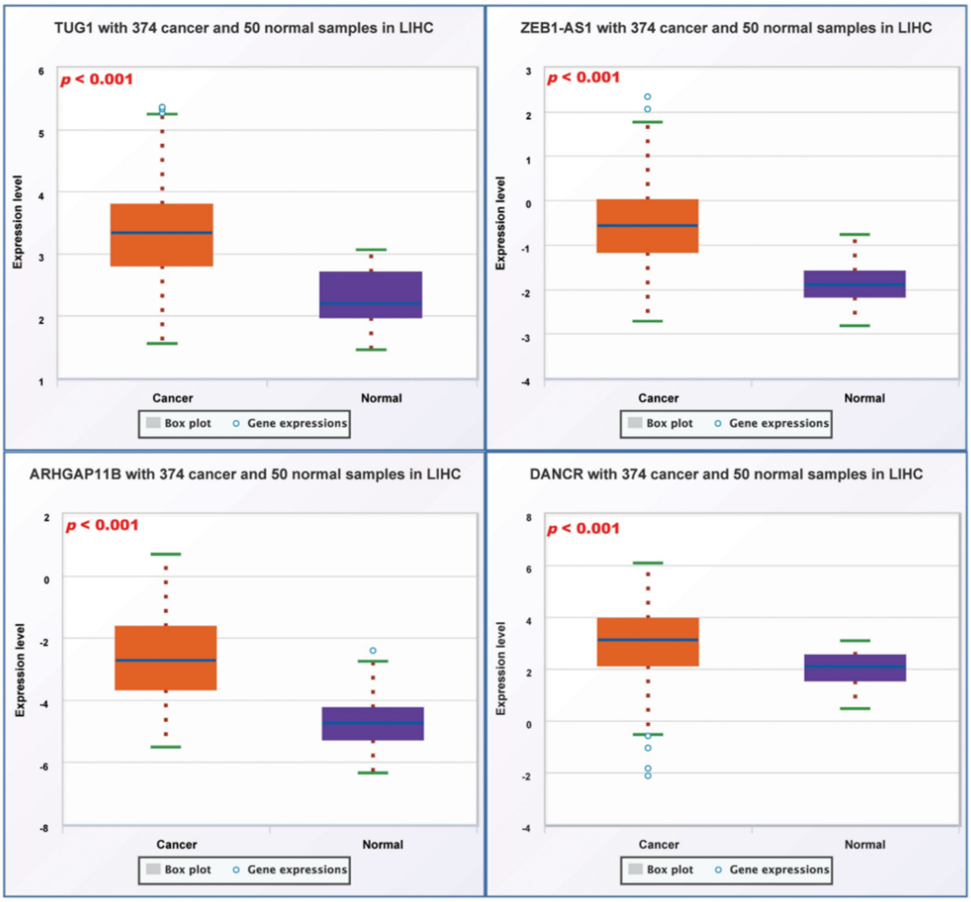
TUG1 with 374 cancer and 50 normal samples in LIHC
p < 0.001
Expression level
Cancer
Normal
Box plot
Gene expressions
ZEB1-AS1 with 374 cancer and 50 normal samples in LIHC
p < 0.001
Expression level
Cancer
Normal
Box plot
Gene expressions
ARHGAP11B with 374 cancer and 50 normal samples in LIHC
p < 0.001
Expression level
Cancer
Normal
Box plot
Gene expressions
DANCR with 374 cancer and 50 normal samples in LIHC
p < 0.001
Expression level
Cancer
Normal
Box plot
Gene expressions

F

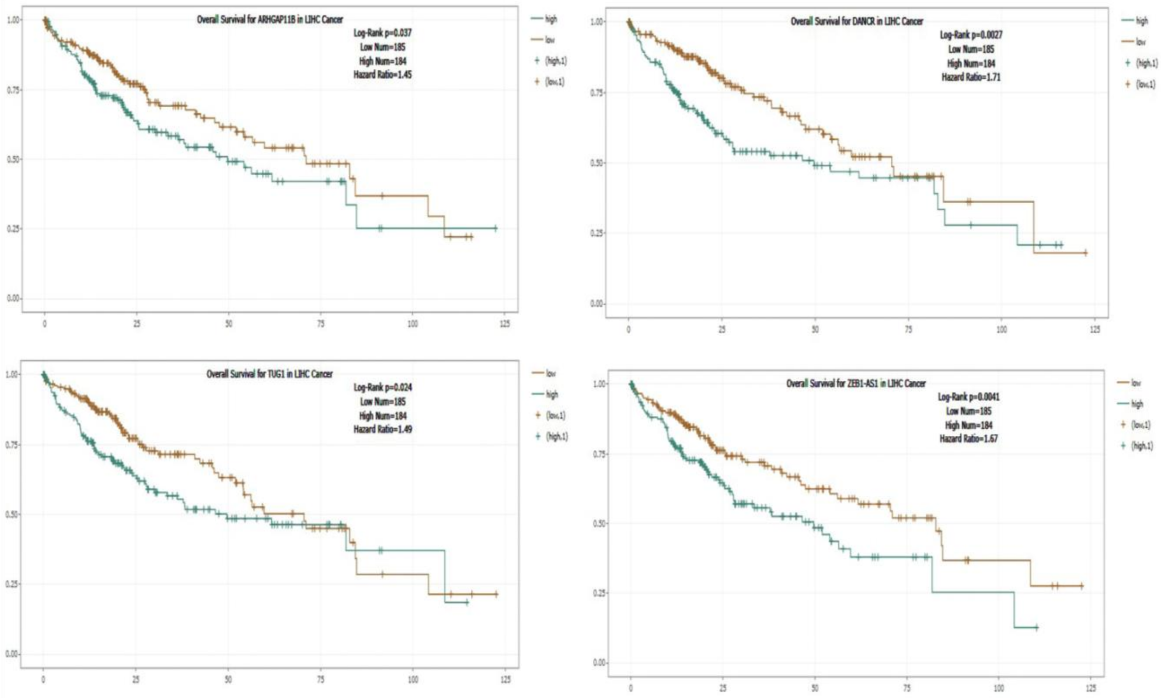
Overall Survival for ARHGAP11B in LIHC Cancer
Log-Rank p=0.037
Low Num=185
High Num=184
Hazard Ratio=1.45
high
low
(high,1)
(low,1)
Overall Survival for DANCR in LIHC Cancer
Log-Rank p=0.0027
Low Num=185
High Num=184
Hazard Ratio=1.71
high
low
(high,1)
(low,1)
Overall Survival for TUG1 in LIHC Cancer
Log-Rank p=0.024
Low Num=185
High Num=184
Hazard Ratio=1.49
low
high
(low,1)
(high,1)
Overall Survival for ZEB1-AS1 in LIHC Cancer
Log-Rank p=0.0041
Low Num=185
High Num=184
Hazard Ratio=1.67
low
high
(low,1)
(high,1)

G

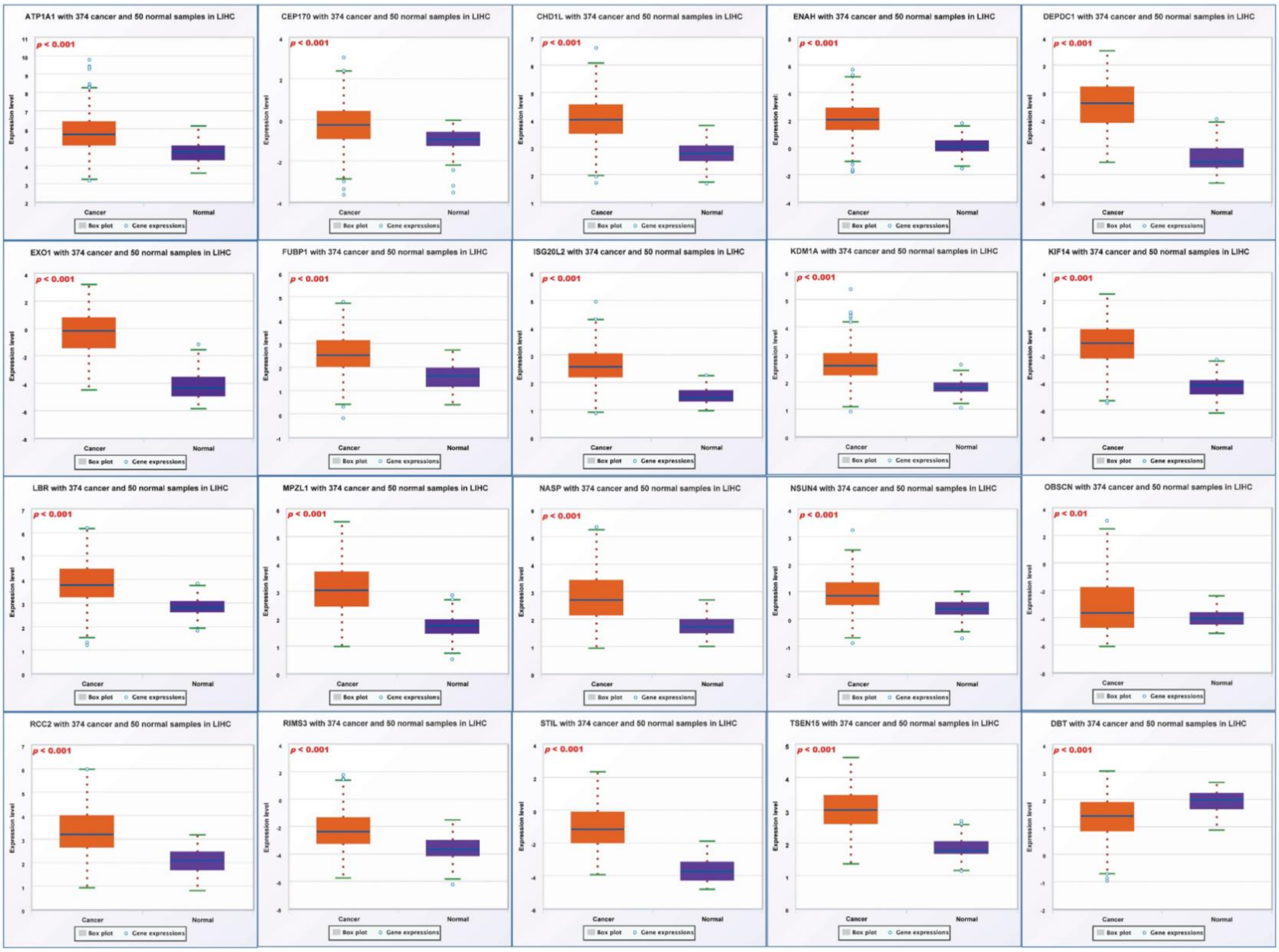

Supplementary Figure S3. ZBTB40-associated with hsa-miR-132-3p network in LIHC. Interaction networks of hsa-miR-132-3p with lncRNAs (A), circRNAs (B), pseudogenes (C), and sncRNAs (D). (E) Expression of hsa-miR-132-3p-associated lncRNAs in LIHC and normal liver tissues. (F) Overall survival of hsa-miR-132-3p-associated with lncRNAs. (G) Expression for hsa-miR-132-3p-associated circRNAs in LIHC. (H) Overall survival of hsa-miR-132-3p-associated circRNAs.

.

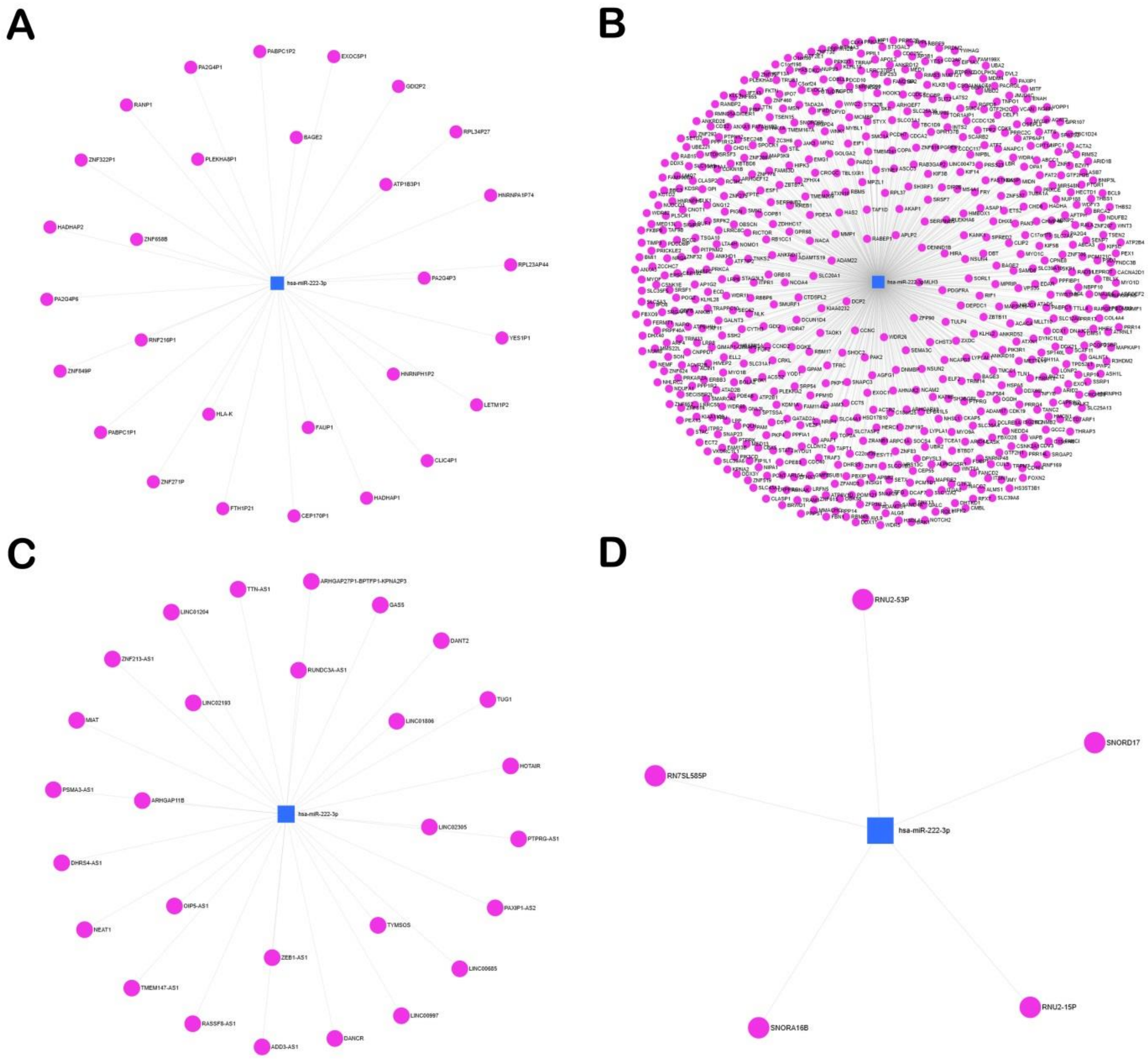

A
hsa-miR-222-3p
PABPC1P2
EXOC5P1
PA2G4P1
GDI2P2
RANP1
BAGE2
RPL34P27
ZNF322P1
PLEKHA8P1
ATP1B3P1
HNRNPA1P74
HADHAP2
ZNF658B
RPL23AP44
PA2G4P3
PA2G4P6
RNF216P1
YES1P1
ZNF849P
HNRNPH1P2
LETM1P2
HLA-K
PABPC1P1
FAUP1
CLIC4P1
ZNF271P
HADHAP1
FTH1P21
CEP170P1
B
C
hsa-miR-222-3p
ARHGAP27P1-BPTFP1-KPNA2P3
TTN-AS1
GAS5
LINC01204
DANT2
ZNF213-AS1
RUNDC3A-AS1
LINC02193
TUG1
MIAT
LINC01806
HOTAIR
PSMA3-AS1
ARHGAP11B
LINC02305
PTPRG-AS1
DHRS4-AS1
OIP5-AS1
PAXIP1-AS2
NEAT1
TYMSOS
ZEB1-AS1
LINC00685
TMEM147-AS1
LINC00997
RASSF8-AS1
DANCR
ADD3-AS1
D
RNU2-53P
SNORD17
RN7SL585P
hsa-miR-222-3p
RNU2-15P
SNORA16B

E

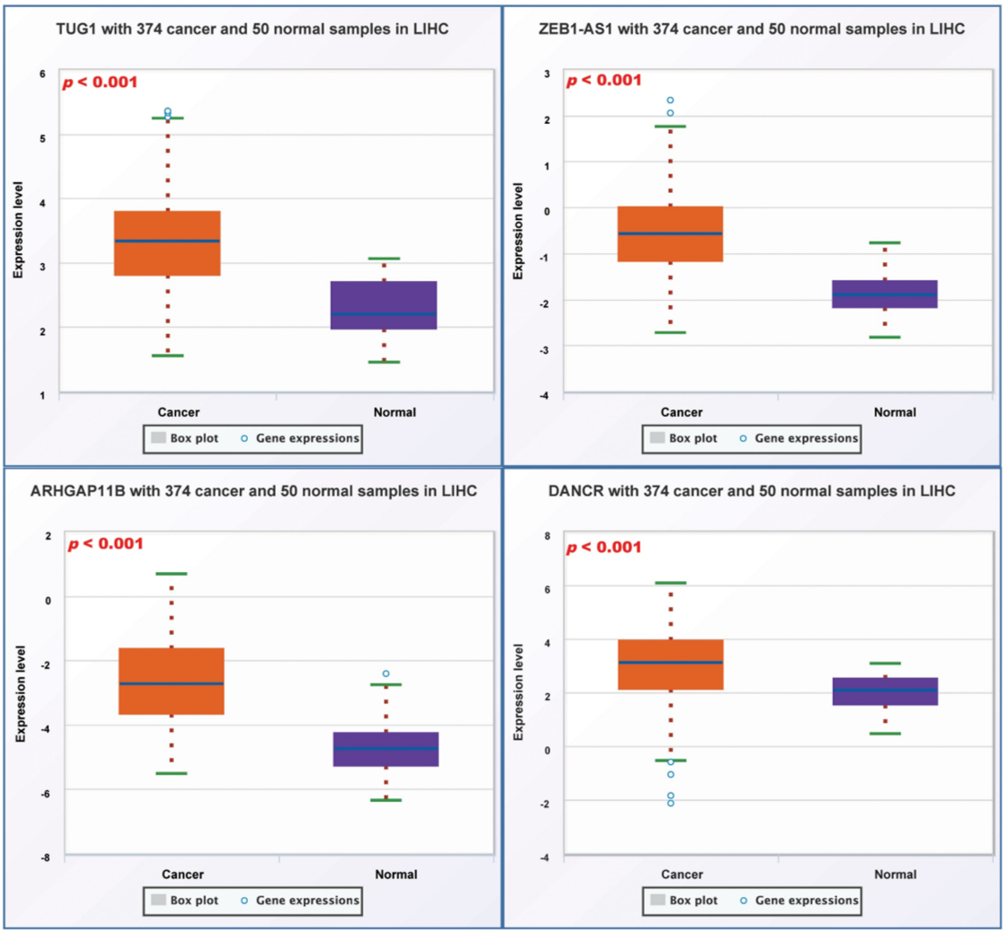

TUG1 with 374 cancer and 50 normal samples in LIHC
p < 0.001
Expression level
6
5
4
3
2
1
Cancer
Normal
Box plot
Gene expressions
ZEB1-AS1 with 374 cancer and 50 normal samples in LIHC
p < 0.001
Expression level
3
2
1
0
-1
-2
-3
-4
Cancer
Normal
Box plot
Gene expressions
ARHGAP11B with 374 cancer and 50 normal samples in LIHC
p < 0.001
Expression level
2
0
-2
-4
-6
-8
Cancer
Normal
Box plot
Gene expressions
DANCR with 374 cancer and 50 normal samples in LIHC
p < 0.001
Expression level
8
6
4
2
0
-2
-4
Cancer
Normal
Box plot
Gene expressions

F

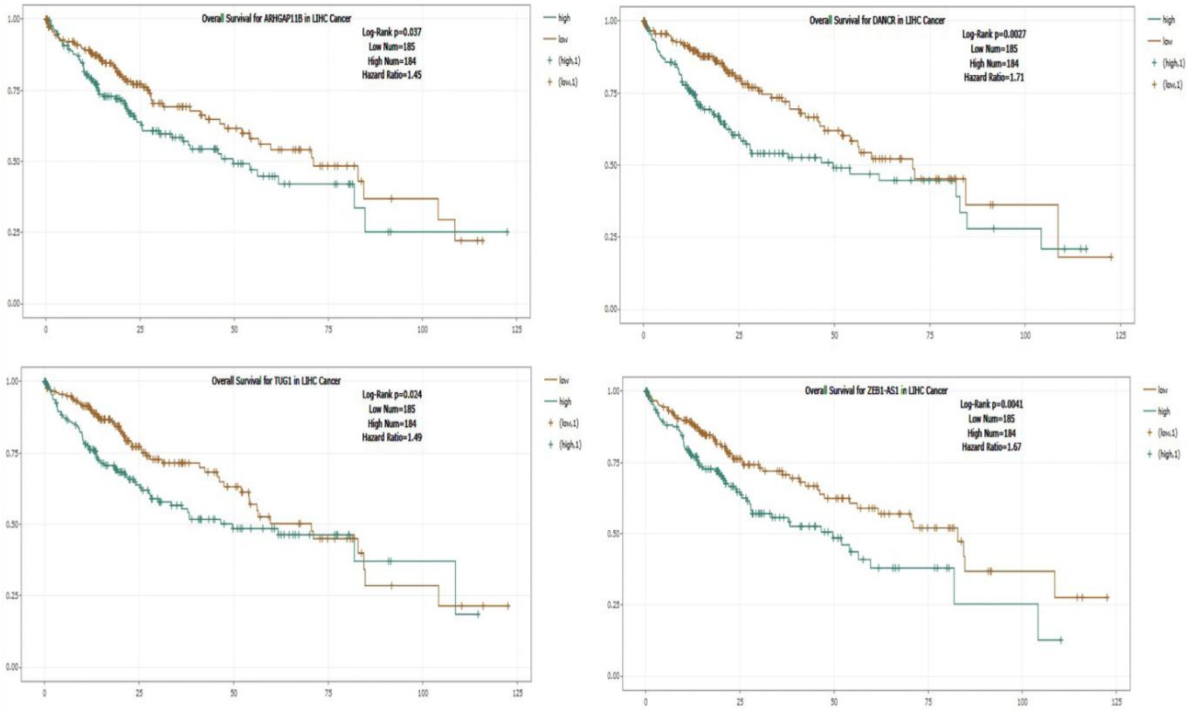

Overall Survival for ARHGAP11B in LIHC Cancer
Log-Rank p=0.037
Low Num=185
High Num=184
Hazard Ratio=1.45
high
low
(high,1)
(low,1)
Overall Survival for DANCR in LIHC Cancer
Log-Rank p=0.0027
Low Num=185
High Num=184
Hazard Ratio=1.71
high
low
(high,1)
(low,1)
Overall Survival for TUG1 in LIHC Cancer
Log-Rank p=0.024
Low Num=185
High Num=184
Hazard Ratio=1.49
low
high
(low,1)
(high,1)
Overall Survival for ZEB1-AS1 in LIHC Cancer
Log-Rank p=0.0041
Low Num=185
High Num=184
Hazard Ratio=1.67
low
high
(low,1)
(high,1)
1.00
0.75
0.50
0.25
0.00
0
25
50
75
100
125

G

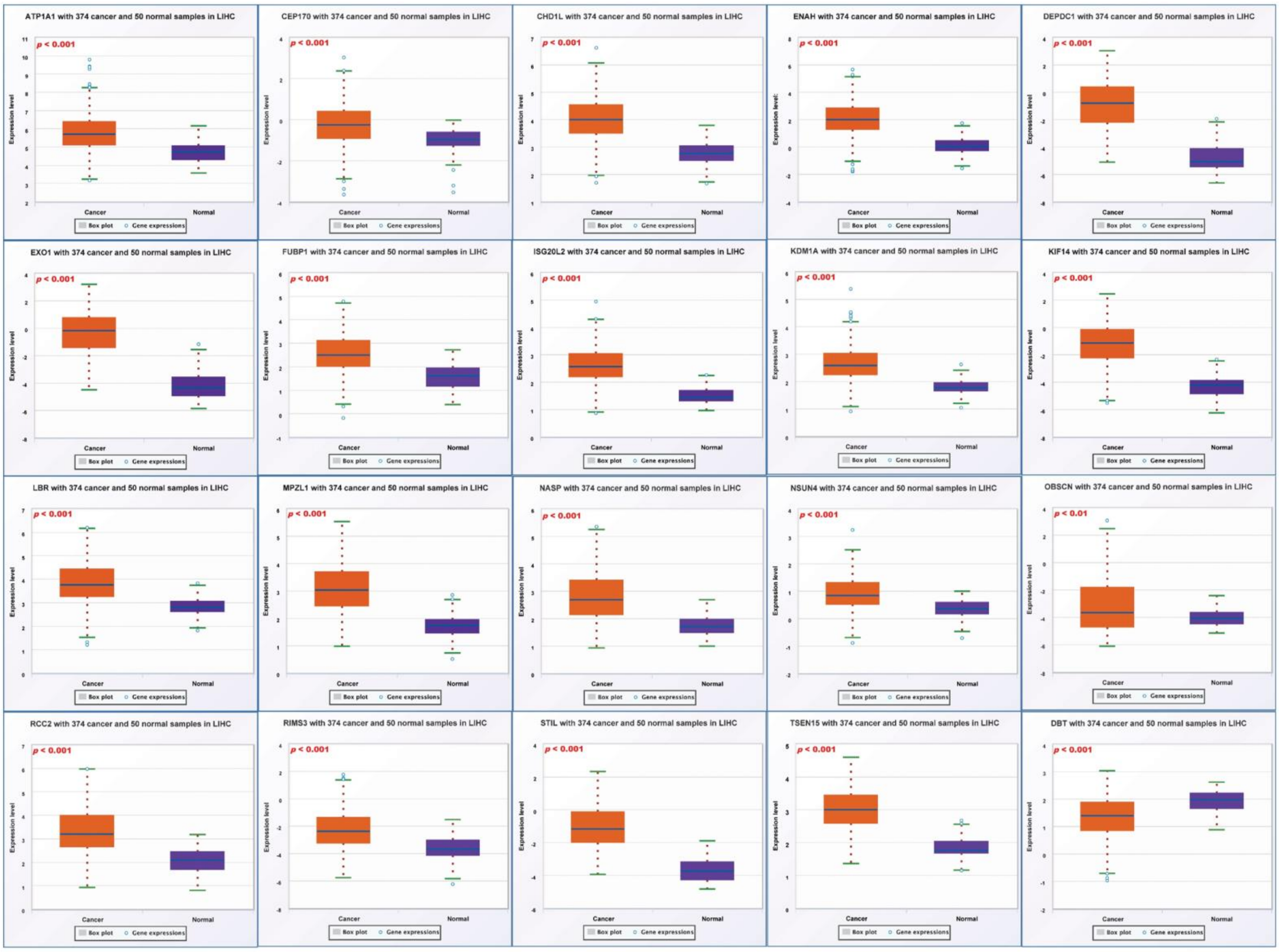
ATP1A1 with 374 cancer and 50 normal samples in LIHC
CEP170 with 374 cancer and 50 normal samples in LIHC
CHD1L with 374 cancer and 50 normal samples in LIHC
ENAH with 374 cancer and 50 normal samples in LIHC
DEPDC1 with 374 cancer and 50 normal samples in LIHC
EXO1 with 374 cancer and 50 normal samples in LIHC
FUBP1 with 374 cancer and 50 normal samples in LIHC
ISG20L2 with 374 cancer and 50 normal samples in LIHC
KDM1A with 374 cancer and 50 normal samples in LIHC
KIF14 with 374 cancer and 50 normal samples in LIHC
LBR with 374 cancer and 50 normal samples in LIHC
MPZL1 with 374 cancer and 50 normal samples in LIHC
NASP with 374 cancer and 50 normal samples in LIHC
NSUN4 with 374 cancer and 50 normal samples in LIHC
OBSCN with 374 cancer and 50 normal samples in LIHC
RCC2 with 374 cancer and 50 normal samples in LIHC
RIMS3 with 374 cancer and 50 normal samples in LIHC
STIL with 374 cancer and 50 normal samples in LIHC
TSEN15 with 374 cancer and 50 normal samples in LIHC
DBT with 374 cancer and 50 normal samples in LIHC
p < 0.001
p < 0.01
Expression level
Cancer
Normal
Box plot
Gene expressions

H

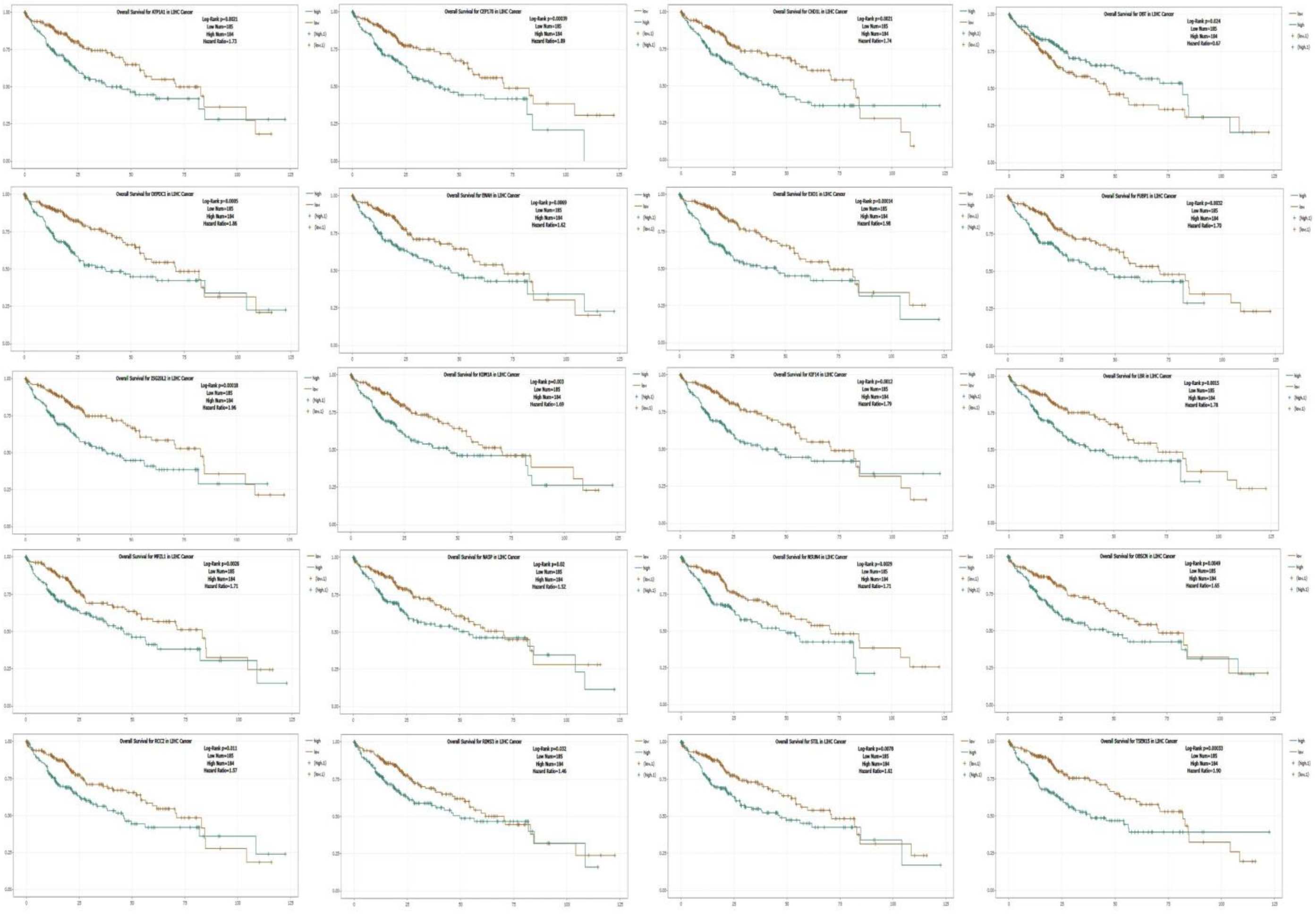

Supplementary Figure S4. ZBTB40-associated with hsa-miR-222-3p network in LIHC. Interaction networks of hsa-miR-222-3p with lncRNAs (A), circRNAs (B), pseudogenes (C), and sncRNAs (D). (E) Expression of hsa-miR-222-3p-associated lncRNAs in LIHC and normal liver tissues. (F) Overall survival of hsa-miR-222-3p-associated with lncRNAs. (G) Expression for hsa-miR-222-3p-associated circRNAs in LIHC. (H) Overall survival of hsa-miR-222-3p-associated circRNAs.

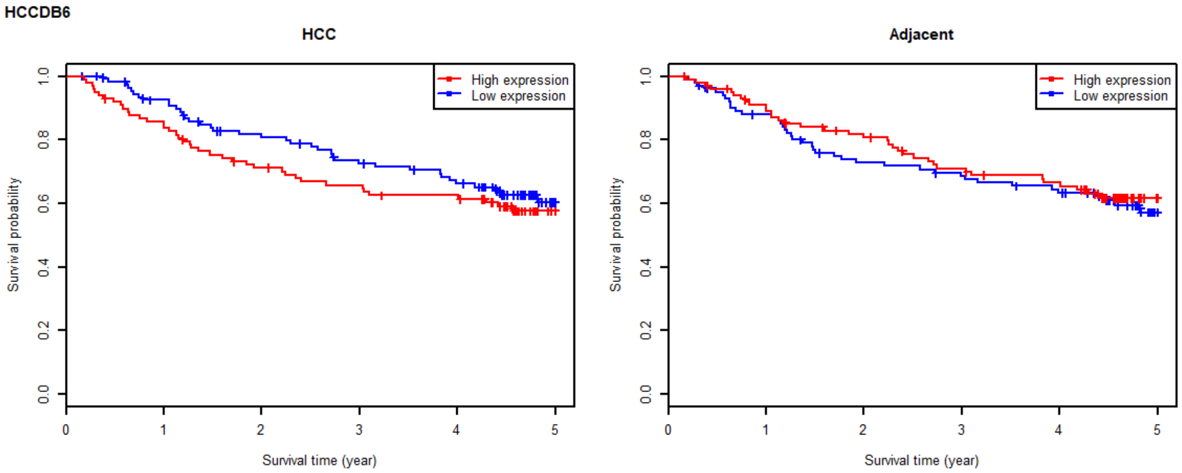

Supplementary Figure S5. External validation of ZBTB40 expression and prognostic relevance in the HCCDB6/GSE14520 cohort.

Table S5. Multivariable Cox regression analysis of ZBTB40 in LIHC (TCGA-LIHC (n=371)).

Variables	Hazard Ratio (HR)	95%CI	P-value
ZBTB40 (High vs Low)	1.682	1.352-2.361	<0.001
Age	1.016	0.999-1.032	0.058
Gender	1.012	0.679-1.507	0.955
Stage (Stage 0 vs Stage I)	0.556	0.352-0.876	0.012
Stage (Stage II vs Stage I)	0.760	0.441-1.310	0.324
Stage (Stage III vs Stage 0)	0.528	0.073-3.830	0.528
